# Supplementary material for: Entropy sorting of single-cell RNA sequencing data reveals the inner cell mass in the human pre-implantation embryo
Source: Stem Cell Reports. 2022 Oct 13;18(1):47–63. doi: 10.1016/j.stemcr.2022.09.007 (PMC9859930; doi:10.1016/j.stemcr.2022.09.007)
Supplement: Document S2. Article plus supplemental information [file mmc2.pdf]

Stem Cell Reports
Article
OPEN ACCESS

Entropy sorting of single-cell RNA sequencing data reveals the inner cell mass in the human pre-implantation embryo

Arthur Radley,[1] Elena Corujo-Simon,[2] Jennifer Nichols,[2] Austin Smith,[3,*] and Sara-Jane Dunn[4,*]
[1]Wellcome-MRC Cambridge Stem Cell Institute, University of Cambridge, Jeffrey Cheah Biomedical Centre, Puddicombe Way, Cambridge Biomedical Campus, Cambridge CB2 0AW, UK
[2]MRC Human Genetics Unit, MRC Institute of Genetics and Cancer, The University of Edinburgh, Western General Hospital, Edinburgh EH4 2XU, UK
[3]Living Systems Institute, University of Exeter, Stocker Road, Exeter EX4 4QD, UK
[4]Microsoft Research, 21 Station Road, Cambridge CB1 2FB, UK
*Correspondence: austin.smith@exeter.ac.uk (A.S.), sjdunn@deepmind.com (S.-J.D.)
https://doi.org/10.1016/j.stemcr.2022.09.007

SUMMARY

A major challenge in single-cell gene expression analysis is to discern meaningful cellular heterogeneity from technical or biological noise. To address this challenge, we present entropy sorting (ES), a mathematical framework that distinguishes genes indicative of cell identity. ES achieves this in an unsupervised manner by quantifying if observed correlations between features are more likely to have occurred due to random chance versus a dependent relationship, without the need for any user-defined significance threshold. On synthetic data, we demonstrate the removal of noisy signals to reveal a higher resolution of gene expression patterns than commonly used feature selection methods. We then apply ES to human pre-implantation embryo single-cell RNA sequencing (scRNA-seq) data. Previous studies failed to unambiguously identify early inner cell mass (ICM), suggesting that the human embryo may diverge from the mouse paradigm. In contrast, ES resolves the ICM and reveals sequential lineage bifurcations as in the classical model. ES thus provides a powerful approach for maximizing information extraction from high-dimensional datasets such as scRNA-seq data.

INTRODUCTION

Single-cell RNA sequencing (scRNA-seq) (Tang et al., 2009) is a powerful technique for studying cell identity and heterogeneity by capturing transcriptome-wide RNA expression at single-cell resolution. As such, scRNA-seq yields an unbiased dataset, rather than being restricted to a predefined subset of genes of interest. However, the cost of this information-rich data is a practical limitation known as the curse of dimensionality (CoD) (Bellman 1967). This phenomenon arises when analyzing datasets with increasingly large dimensions: the number of features or variables. In the context of scRNA-seq, we typically refer to each gene as a feature and each cell as sample. As the number of features increases, our ability to discern patterns between samples and/or features decreases (Altman and Krzywinski 2018). Thus, by sequencing tens of thousands of genes, we may reduce our ability to identify differential gene expression patterns. The challenge is exacerbated by technical artifacts introduced during data collection, such as batch effects and false negative dropouts (Kiselev et al., 2019), which weaken the correlations between cells and genes.

The antidote to this challenge is the blessing of dimensionality (Zimek et al., 2012): if the features within a dataset are highly structured, so their values correlate strongly, the presence of additional correlated features will increase our ability to separate distinct samples. This implies that the CoD may be viewed as the presence of a large number of features whose values are random in relation to groups of similar samples. In scRNA-seq, such features correspond to genes that do not inform cell state, such as housekeeping genes. It has been estimated that of the tens of thousands of distinct transcripts captured in a typical scRNA-seq assay, only 3,000–5,000 of them relate to cell-type-specific expression patterns (Ramskö Ld et al., 2009).

To overcome the high dimensionality of scRNA-seq data, several methodologies have been developed (Kiselev et al., 2019; Wu and Zhang 2020). The most commonly used are feature extraction and highly variable gene (HVG) selection. Feature extraction methods such as principal component analysis and uniform manifold approximation and projection (UMAP) (McInnes et al., 2018) attempt to compress a high-dimensional dataset into a smaller set of highly informative features. HVG selection seeks to identify a subset of genes more predictive of distinct cell types than randomly expressed genes. While it is a widely used pre-processing strategy, HVG selection can struggle to account for important but lowly expressed genes or genes present in only a small fraction of cells (Källberg et al., 2021). Furthermore, evaluation of various HVG methods found that different techniques show poor overlap in HVGs suggested from the same datasets and that highly expressed genes were often incorrectly flagged as HVGs (Yip et al., 2018). This poor consistency may arise because gene selection is carried out in a univariate manner based on a weak mechanistic assumption that genes with high expression variance correspond to different cell types.

This is an open access article under the CC BY license (http://creativecommons.org/licenses/by/4.0/).

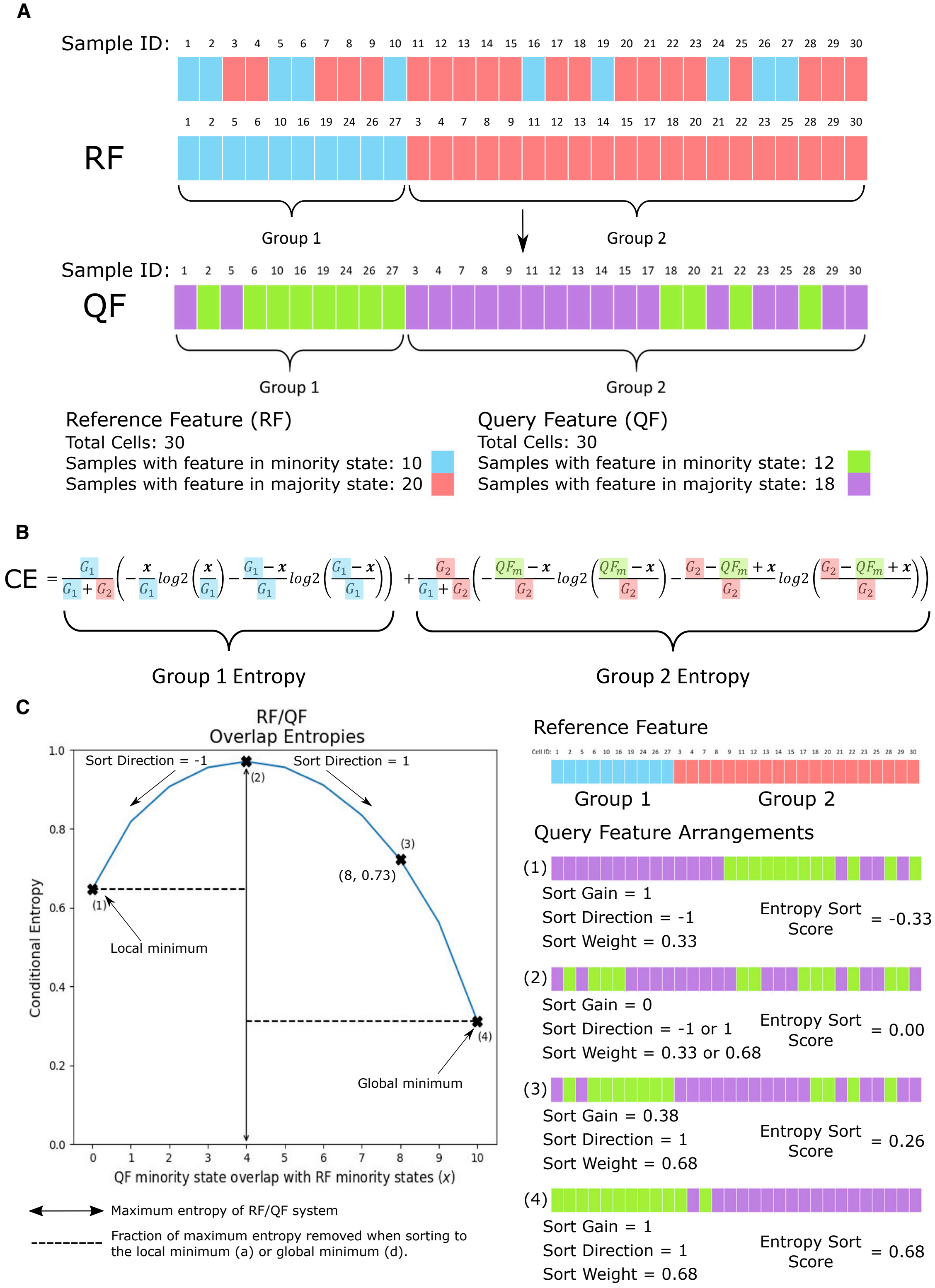

$$CE = \frac{G_1}{G_1+G_2}\left(-\frac{x}{G_1}log2\left(\frac{x}{G_1}\right)-\frac{G_1-x}{G_1}log2\left(\frac{G_1-x}{G_1}\right)\right)+\frac{G_2}{G_1+G_2}\left(-\frac{QF_m-x}{G_2}log2\left(\frac{QF_m-x}{G_2}\right)-\frac{G_2-QF_m+x}{G_2}log2\left(\frac{G_2-QF_m+x}{G_2}\right)\right)$$

Figure 1. Quantifying the dependent relationship between two features

(A) A toy example. The states of each RF sample are sorted into two groups. The QF is then inspected while maintaining the RF sample ordering.

(legend continued on next page)

In this work, we introduce a mathematical framework termed entropy sorting (ES). ES allows us to simultaneously measure the correlations between features while quantifying the likelihood that these correlations have been weakened due to the introduction of technical error, such as dropouts. We encode ES in an algorithm called FFAVES: functional feature amplification via entropy sorting. We use FFAVES to amplify the signal of groups of co-regulating genes in an unsupervised, multivariate manner. By amplifying the signal of genes with correlated expression, while filtering out genes that are randomly expressed, we can identify a subset of genes more predictive of different cell types. The output of FFAVES can then be used in our second algorithm, entropy sort feature weighting (ESFW), to create a ranked list of genes that are most likely to pertain to distinct sub-populations of cells in an scRNA-seq dataset. Unlike HVG selection, ESFW performs gene selection in a multivariate manner that specifically seeks to identify genes with consistent expression patterns, indicative of a distinct cellular identity.

RESULTS

Entropy sorting

Pairwise feature correlations

The foundation of ES is to create a correlation metric analogous to metrics such as Pearson's correlation or mutual information to define a common structure between the discrete states of two features. To do so, we re-imagine conditional entropy (*CE*) as a sorting problem between features whose samples can display two states, e.g., a gene being functionally active or inactive. As such, discretization of continuous data is a requirement for ES (supplemental information section 1). Given two features, $CE \in [0, 1]$ quantifies the information needed to predict the state of one feature for a given sample when conditioned on the other feature. For example, $CE = 0$ indicates that the observed state of the conditioned feature is entirely determined upon observing the state of the second feature.

To develop *CE* into a sorting problem, we consider a toy example to guide the theoretical exposition (Figure 1A) in which 30 samples (cells) display discrete states for two features (genes). We hypothesize that partitioning all samples by the observed states of one feature will perfectly sort the states of the other. We designate the partitioning feature as the reference feature (RF). We then seek to quantify to what degree the RF sorts the states of the second feature, the query feature (QF). We calculate *CE* for the RF/QF pair via the entropy sort equation (ESE, Figure 1B). A detailed derivation of the ESE is provided in supplemental information section 2. Partitioning samples according to the RF form two groups (G_I , $i = 1, 2$, Figure 1A). We can calculate the entropy of each group as a function of the number of QF minority states in G_1: $QF_{m,G1}$ (hereafter denoted as x).

The ESE defines a smooth parabolic function for calculating $CE(x)$. We plot the ESE parabola for our toy example in Figure 1C and highlight four points of interest on the curve. Points (1) and (4) are the boundaries of the ESE, since $x \geq 0$ (1) and $x \leq G_1$ (4). Point (2) corresponds to the maximum *CE*, the point at which the RF/QF pair is independent. Point (3) is *CE* of the observed samples in Figure 1A. The ESE parabola represents a common structure for any RF/QF pair that we use to define our correlation metric, which is important for two reasons. It demonstrates that any RF/QF pair, regardless of sample number or minority/majority state cardinality, has a quantifiable common structure. Further, it defines a mathematical framework that can be used to calculate the relationship between two features under the assumption that the features are dependent upon one another. Later we consider the assumption of feature independence, to quantify which of the two hypotheses is more likely.

The ES correlation metric can be broken down into three parts: sort direction (SD), sort weight (SW), and sort gain (SG).

SD describes whether the value of x corresponds to an enrichment of QF minority states in G_1 or G_2, such that

$$\begin{aligned} &\text{If } x < x_{maximum},\ SD = -1, \\ &\text{Else if } x > x_{maximum},\ SD = 1, \end{aligned} \quad \text{(Equation 1)}$$

where $x_{maximum}$ is the value of x at the maximum of the parabola. If SD = 1, the system is sorting toward the global minimum, since the minimum when $x > x_{maximum}$ always has a lower *CE* than when SD = −1. SW is the maximum amount of entropy that would be removed from the system

(B) The ESE for calculating *CE*. G_1 (group 1) and G_2 (group 2) are the number of minority or majority states of the RF respectively. QF_m is the total number of QF minority states. For brevity, we use x to denote $QF_{m,G1}$, the number of QF minority states that overlap with the RF minority states, which is the only independent variable. Each constant is highlighted with their corresponding colors in (A).
(C) Given any observed pair of features, we may form an ESE parabola by fixing the constants of the ESE and calculating the *CE* for different values of x. Points (1) and (4) correspond to the local and global minimum. (2) is the maximum *CE*, where the RF and QF are independent. (3) is the *CE* corresponding to the observed arrangement in (A). Each of (1)–(4) is illustrated by an example arrangement.

if it existed at either minimum. Hence, it is dependent on SD:

$$SW = \begin{cases} \dfrac{MaxEnt - MinEnt_{local}}{MaxEnt}, & \text{if SD} = -1, \\ \dfrac{MaxEnt - MinEnt_{global}}{MaxEnt}, & \text{if SD} = 1, \end{cases}$$

(Equation 2)

where $MaxEnt$, $MinEnt_{local}$ and $MinEnt_{global}$ are the maximum, local and global minimums defined by the ESE parabola.

Lastly, SG is similar to a well-established metric from information theory: information gain (IG) (Quinlan 1986). IG refers to the decrease in entropy from the scenario in which QF and RF are independent to when QF is dependent on RF. SG is the same decrease in entropy, but as a fraction of the total entropy that would be removed at the relevant minimum. Formally,

$$SG = \begin{cases} \dfrac{MaxEnt - ObsEnt}{MaxEnt - MinEnt_{local}}, & \text{if SD} = -1, \\ \dfrac{MaxEnt - ObsEnt}{MaxEnt - MinEnt_{global}}, & \text{if SD} = 1, \end{cases}$$

(Equation 3)

where $ObsEnt$ is the observed entropy for a given RF/QF pair (point (3) in Figure 1C).

The entropy sort score (ESS) is the product of these values for any RF/QF pair:

$$ESS = SD \times SW \times SG,$$

(Equation 4)

where $SD = \pm 1$, $0 \le SG \le 1$ and $0 \le SW \le 1$. Hence, for any RF/QF pair, $ESS \in [-1, 1]$, similar to other correlation metrics.

Divergence

The ESE parabola describes how CE changes for an RF/QF system with fixed minority/majority state proportions but varying RF/QF dependencies. To quantify to what degree observed RF/QF samples appear to be in the wrong state, we introduce divergence: a measure of how far away the observed states of an RF/QF pair are from an optimally dependent system.

$$Divergence = \begin{cases} ObsEnt - MinEnt_{local}, & \text{if SD} = -1, \\ ObsEnt - MinEnt_{global}, & \text{if SD} = 1. \end{cases}$$

(Equation 5)

To demonstrate the concept of divergence, we consider three toy examples (Figures 2Ai–2Aiii). Example (i) represents the ground truth, where a strong but imperfect dependent relationship exists between the observed states of the RF/QF pair. Here we observe the maximum number of minority state RF/QF sample overlap, indicated by the samples that have blue and green expression states ($x = 10$). Accordingly, CE is equal to the global minimum of the ESE parabola. In (ii) we introduce an error such that one of the minority state QF samples is incorrectly observed as a majority state (marked "X"). This changes the parameters of the ESE, causing the parabola to shift (blue to orange). Since the error occurs in the non-overlapping region of the RF/QF minority states (the green minority state where the dropout occurred does not overlap with a blue minority state in the ground truth), the observed system still exists on the global minimum, and no divergence has been observed. In example (iii), we introduce an erroneous data point in the overlapping minority state samples. This does not alter the parameters of the ESE, so the parabola is unchanged (orange), but the observed CE is greater than the global minimum. This movement away from the global/local minimum ($SD = 1/-1$) is the divergence, calculated by Equation 5.

The simple examples in Figure 2A introduce scenarios where error can be observed through divergence. We have identified eight distinct scenarios in which error in an RF/QF system may be observed through divergence (Figure S1). Distinguishing between these scenarios is important for the implementation of ES, as discussed later.

ES hypothesis testing

We use the concept of divergence to perform hypothesis testing. We test both the hypothesis that the features are dependent, so observed divergence is due to technical noise/error, and the null hypothesis that the features are independent.

Under the assumption of dependence, the ESE quantifies the distance an observed RF/QF pair is from optimal dependence. We consider each divergent state, a state that moves the observed system away from the local/global minimum. We assign a proportion of the total divergence to each cell showing a divergent state, as the divergence per cell (DPC),

$$DPC_{Dependent} = \begin{cases} \dfrac{ObsEnt - MinEnt_{local}}{\text{Number of divergent cells}}, & \text{if SD} = -1, \\ \dfrac{ObsEnt - MinEnt_{global}}{\text{Number of divergent cells}}, & \text{if SD} = 1. \end{cases}$$

(Equation 6)

When $SD = 1$, divergent cells are those where an RF minority state overlaps with a QF majority state (Figures 2Aiii and 2Biv). When $SD = -1$, divergent cells occur when an RF minority state overlaps with a QF minority state. We visualize the DPC for example (iv) in Figure 2Biv as the gradient between the global minimum and the observed entropy (green line).

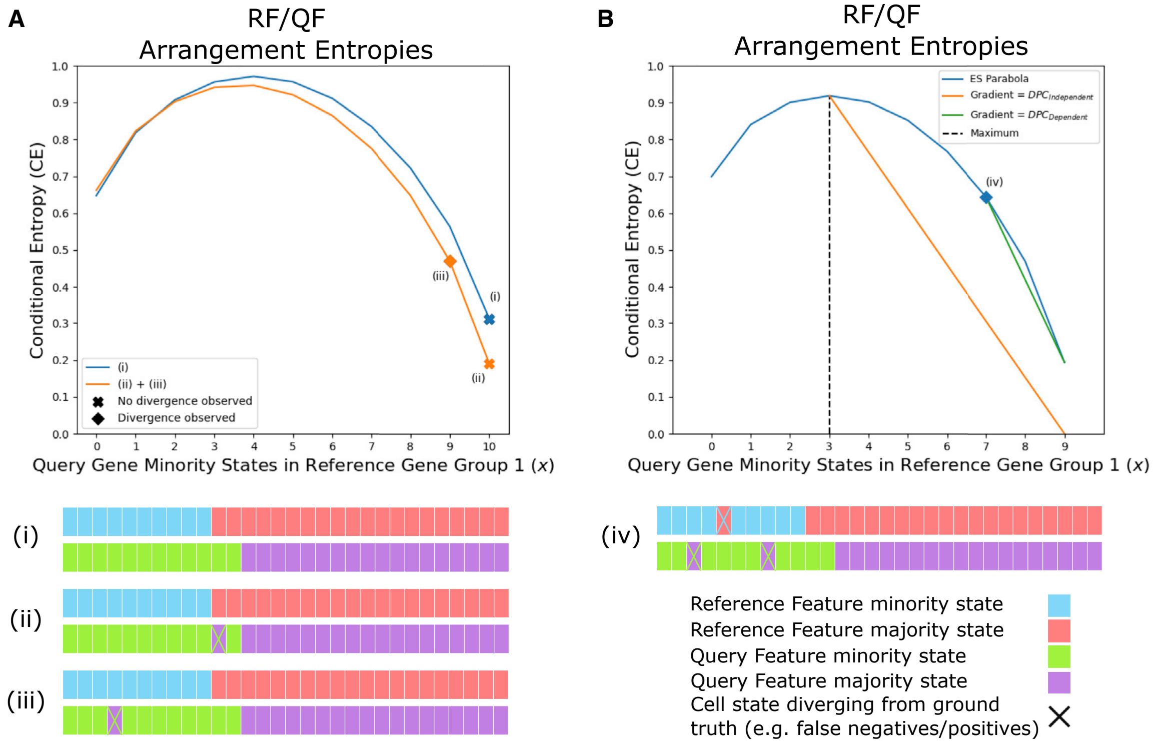

Figure 2. ES divergence and error potential

(A) ESE parabolas highlighting three toy examples to demonstrate divergence: (i) ground truth for a partially dependent system; (ii) an FN dropout added that does not produce observable divergence on the ESE parabola; (iii) the addition of an FN dropout that generates observable divergence.

(B) DPC (Equation 6) introduced to the RF/QF pair due to erroneous data points (example iv) under both the assumption of either RF/QF dependence (green line) or independence (orange line).

Under the null hypothesis, we assume RF/QF is independent and the observed minority state overlap (x) has occurred by chance. Therefore, after a sufficient number of re-samplings of observed states from both RF and QF, we would expect on average that x and CE would equal their values at the maximum of the ESE parabola. Hence, under the null hypothesis,

$$DPC_{Independent} = \begin{cases} \dfrac{Maximum\ CE}{x_{Maximum} - x_{Local\ Minimum}} & if\ \ SD = -1, \\ \dfrac{Maximum\ CE}{x_{Global\ Minimum} - x_{Maximum}} & if\ \ SD = 1. \end{cases}$$

(Equation 7)

In Figure 2B, $DPC_{Independent}$ is the gradient of the orange line.

We combine $DPC_{Dependent}$ and $DPC_{Independent}$ to define our final metric, the error potential (EP). EP allows us to compare whether observed divergence is more likely due to the hypothesis that RF and QF are dependent and error has been introduced to the system, or instead that the features are independent:

$$EP = DPC_{Dependent} - DPC_{Independent}.$$

(Equation 8)

$EP > 0$ indicates that hypothesis of dependence holds, while $EP < 0$ indicates that the null hypothesis holds. In our software, we use EP to minimize the presence of erroneous data points and, in turn, amplify the signal of features that have dependent relationships. For information regarding how to identify which feature is the RF and QF for any pair, please see supplemental information section 3.

FFAVES and ESFW

The metrics defined by ES are encoded in two algorithms. The first, FFAVES, uses ES to identify data points in a discrete matrix that are statistically likely to be displaying the wrong state. By correcting these data points, we aim to amplify the signal of feature correlations. The second algorithm, ESFW, assigns an importance weight to each feature in the data. Higher weights indicate that a feature is more likely to belong to a set of dependent features, while

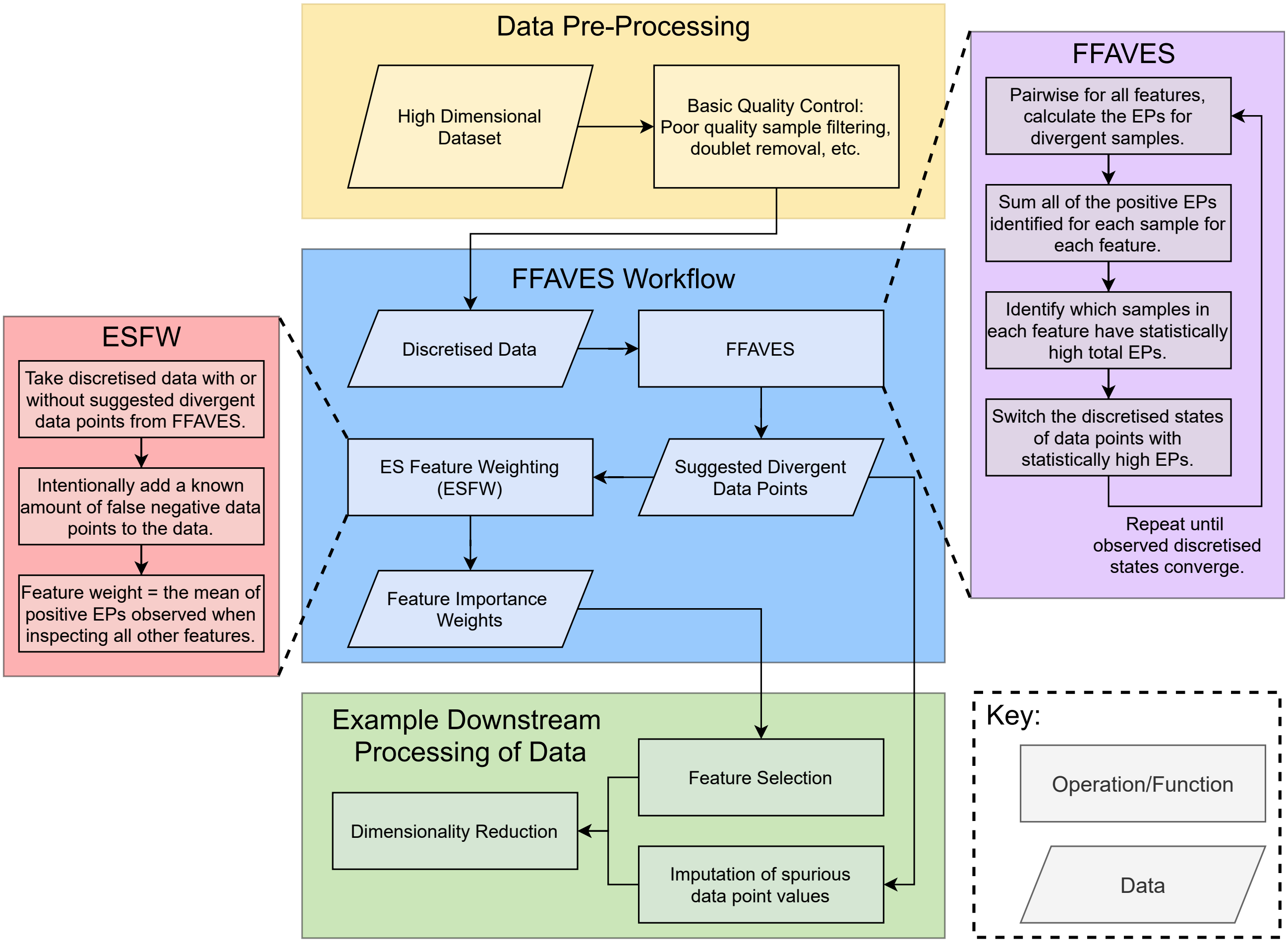

Figure 3. FFAVES and ESFW workflow

Yellow, blue, and green boxes provide the proposed workflow to apply FFAVES and ESFW to high-dimensional data for unsupervised feature selection. The purple and red boxes outline each algorithm.

lower weights pertain to features that are randomly expressed throughout the data.

FFAVES and ESFW encode the mathematical framework of ES to perform multivariate expression state correction and feature importance weighting, respectively. EP is the cornerstone of both software, enabling unsupervised analysis while identifying combinatorial gene expression patterns. EP implicitly identifies whether the relationship between any two features is more likely to have occurred by chance or due to some functional relationship, without any need to define a threshold for this decision. This simultaneously allows the software to ignore uninformative pairs of genes in a manner that directly addresses the CoD. To the authors' knowledge, there are no alternative unsupervised multivariate feature selection techniques available that are specialized for bioinformatics (Saeys et al., 2007).

Figure 3 provides a workflow for FFAVES and ESFW, while detailed descriptions of each software are found in supplemental information sections 4 and 5. We emphasize that the aim of FFAVES and ESFW is to identify genes that are consistently co-expressed within a scRNA-seq dataset. This does not necessarily constitute all genes that have unique expression patterns within distinct cell populations. Some genes may be missing due to technical limitations or poor discretization. Rather, by filtering to ES selected genes, we amplify the predominant expression structure in the data. Subsequently, users should consider how other genes may relate to the refined resolution of cell populations. This is illustrated below, in our analysis of human pre-implantation embryo data.

Synthetic data

We curated a simple synthetic dataset with known ground truth to quantify the performance of FFAVES and ESFW (Figure 4A, with further detail in supplemental information section 7). Briefly, we define five synthetic cell types

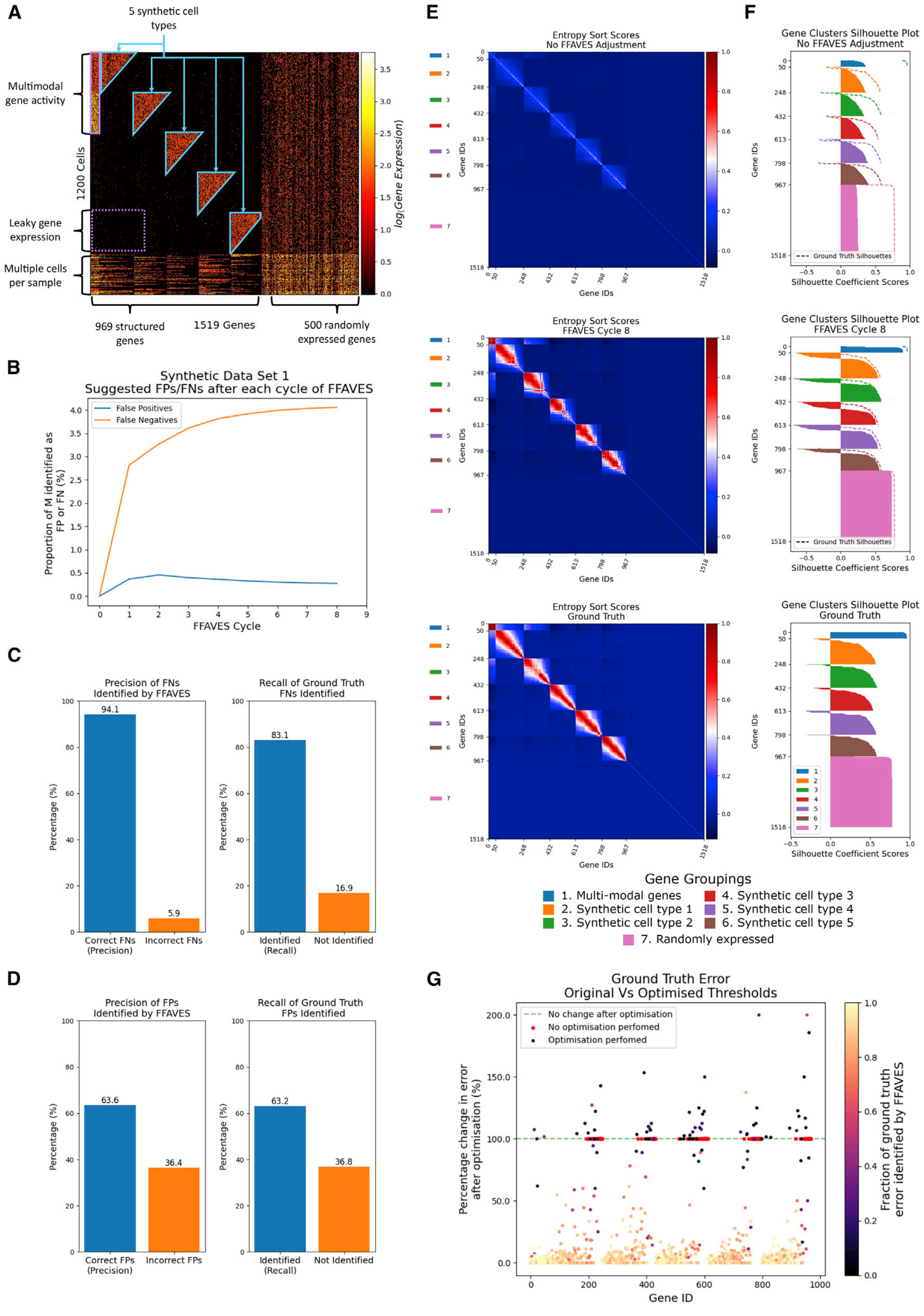

(legend on next page)

according to sets of ~200 "highly structured" genes that are tightly and uniquely co-expressed (Figure 4E, bottom panel). Subsequently, we introduce random dropouts to these cells to mimic technical error (Figure 4E, top panel). We include an additional 500 randomly expressed genes that are designated as "uninformative" genes, as well as "leaky gene expression," which refers to false positive (FP) expression of highly structured genes outside of a gene's specific cell type. We introduce 50 multi-modal genes that have a "medium" expression level in one cell type and "high" in a second. Finally, we created a set of multiple cells (20% of all cells) by randomly combining expression profiles from the five synthetic cell types.

We use this relatively simple representation of scRNA-seq data to dissect the performance and limitations of FFAVES and ESFW, as it facilitates a level of detailed analysis that could not be obtained from a dataset generated by stochastic simulation. To demonstrate the generalizability of ES we further apply ES to synthetic data generated by the Dyngen scRNA-seq simulation software (Cannoodt et al., 2021), as shown in Figures S2, S3, and S4.

FFAVES accurately identifies FN and FP data points

We apply FFAVES to our first synthetic dataset to illustrate how the algorithm amplifies co-regulatory patterns of the 969 structured genes in an unsupervised, multivariate manner. First, to discretize the data, we sampled a discretization threshold from an N (4, 0.2) distribution for each gene. Since the mean expression for each gene is 5, these thresholds increase the probability that a cell in which the gene has nonzero expression (Figure 4A) will display that gene as inactive in the discrete matrix. This producedFNs caused by sub-optimal discretization strategies, which we introduce intentionally to demonstrate that FFAVES can automatically account for such problems.

After eight cycles of FFAVES, the system converges on a set of suggested FN/FP data points (Figure 4B). We can then compare the discrete expression matrix output by FFAVES with the ground truth matrix (Figure S4A). FFAVES performs well, with precision and recall scores of 94.1% and 83.1%, respectively, for the identification of FNs, and 63.6% and 63.2% for FPs (Figures 4C and 4D). We verified that these precision and recall scores are robust across repeated stochastically generated synthetic datasets (Figure S5).

The majority of FNs that FFAVES fails to identify occur in genes active in very few cells, even in the ground truth data with no intentional FNs. This represents a limit of sensitivity where genes expressed in roughly 20 or fewer samples are difficult for FFAVES to repair (supplemental information section 6). Although there is a drop in performance for FP identification, FFAVES is designed to be intentionally conservative when suggesting FP data points to maximize the accuracy of FN identification (supplemental information section 4). Furthermore, the authors are unaware of an existing methodology that can discriminate between FN and FP data points. We also find that FFAVES does not suggest that any of the 500 randomly expressed genes contained FNs or FPs. This is because ES hypothesis testing can discriminate between overlapping minority states between independent and dependent features.

In Figure 4E, we show the ESS (Equation 4) for all gene pairs to illustrate the recovery of gene co-expression relationships. We compare the synthetic data with dropouts (top) with after the application of FFAVES (middle) and the ground truth (bottom). Figure 4F corroborates Figure 4E, showing that the silhouette scores for the seven main gene expression groups are corrected to values close to those of the ground truth. The small groups of genes with high negative scores (Figure 4F, middle) are the same genes as previously highlighted, which are expressed in a small number of cells, so FFAVES struggles to recover ground truth co-expression patterns. These genes have negative silhouette scores because their expression profiles are now more similar to randomly expressed genes than the repaired highly correlated genes. Hence the negative scores of genes in clusters 2–6 indicate that they should be part of randomly expressed genes cluster (pink), rather than their ground truth cluster.

Finally, Figure 4G demonstrates the ability of FFAVES to automatically account for sub-optimal discretization thresholds. Controlled identification of such FNs is equivalent to correcting the discretization threshold. Since we know which FNs resulted from the sub-optimal discretization process, we can quantify the proportion that were corrected by FFAVES. For our synthetic dataset, FFAVES correctly identified 83.1% of these FNs. Once again, a large proportion of the 16.9% FNs not identified correspond to genes for which

Figure 4. FFAVES accurately identifies false negatives and false positives

(A) The synthetic scRNA-seq dataset.

(B) Convergence of FN/FP data points identified after each cycle of FFAVES.

(C and D) Precision and recall scores of FNs and FPs identified by FFAVES, respectively.

(E) Heatmaps of pairwise feature ESSs. Top: before identification of FNs and FPs by FFAVES. Middle: after application of FFAVES. Bottom: ground truth, i.e., synthetic data prior to introduction of FN dropouts.

(F) Silhouette scores of the seven main gene groups calculated from the respective ESSs in (E). Dashed lines outline the ground truth silhouette scores.

(G) Reduction in FN errors that were intentionally introduced by sub-optimal feature discretization.

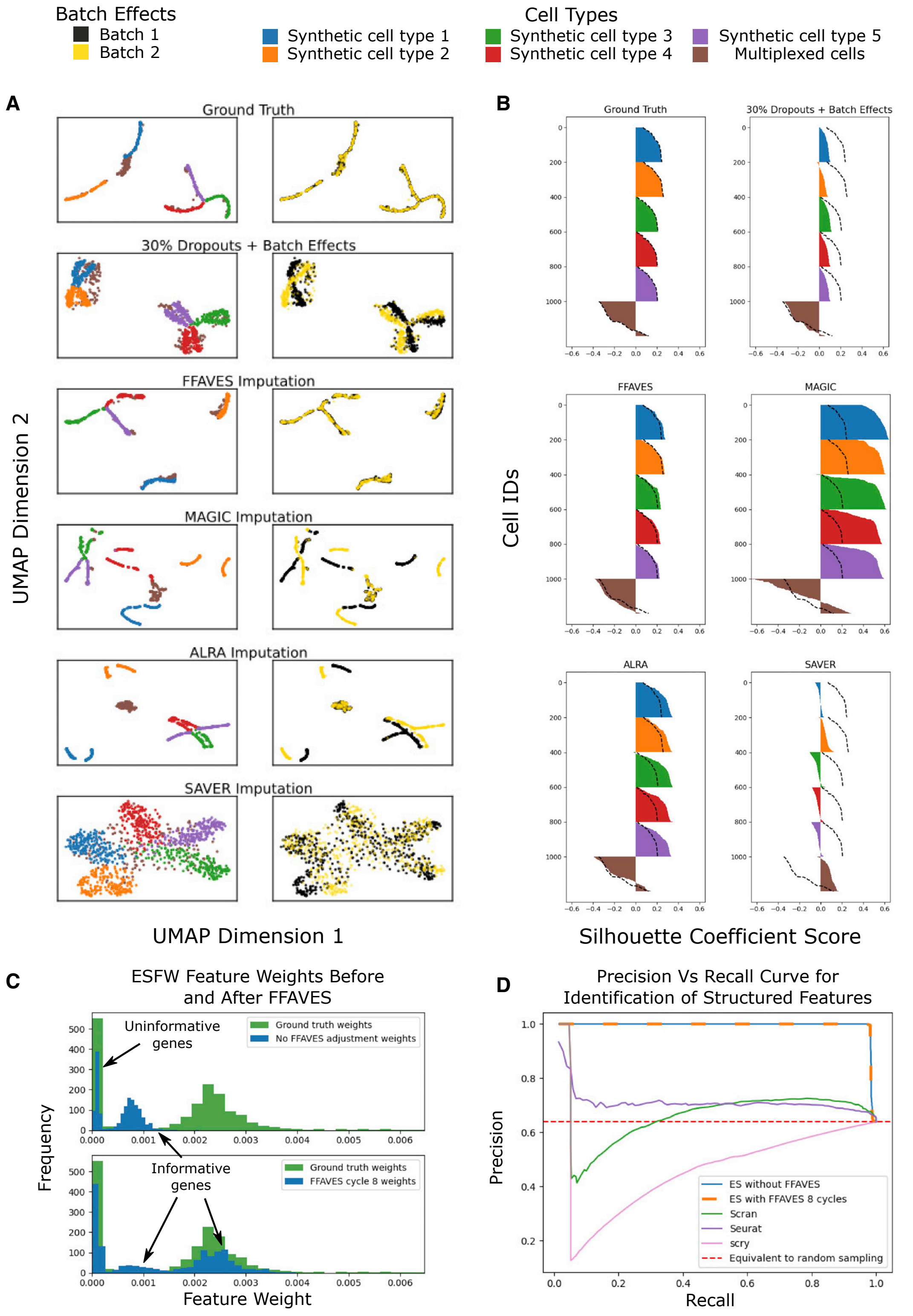

Figure 5. Performance of FFAVES and ESFW against comparable software

(A) UMAPs of the synthetic dataset before and after imputation. The top two plots show the synthetic data before and after FN dropouts were introduced, with no imputation.

(legend continued on next page)

the number of cells with active expression was below the sensitivity of FFAVES (Figure 4G, dark or red markers).

FFAVES facilitates accurate FN imputation

To compare against other tools designed to repair FN data points, we used the FNs suggested by FFAVES to perform imputation. To focus on quantifying correctly identified FNs, we chose a simple method for estimating the values of suggested FNs, the *impute.IterativeImputer* function from the *sklearn* Python package. This yields an imputed gene expression matrix for comparison with other scRNA-seq imputation software: MAGIC (Dijk et al., 2018), ALRA (Linderman et al., 2022), and SAVER (Huang et al., 2018). These tools were chosen as top-ranking examples of software that cover the three main classes of imputation methods (Hou et al., 2020): smoothing (MAGIC), low-rank matrix-based approximation (ALRA), and probabilistic modeling (SAVER).

Figure 5A uses UMAPs to visualize how well each imputation method repairs the synthetic data with dropouts and batch effects. Qualitatively, FFAVES performs best, yielding an embedding most similar to the ground truth. The most notable difference in the MAGIC and ALRA embeddings is the amplification of batch effects, observed as tight groups of the same cell types separated by batch (yellow and black clusters). When the ground truth is known, identification of spurious imputations such as batch effect amplification is easy. However, without a ground truth, identifying spurious imputation becomes more difficult. This can lead to clusters of cells that are computational artifacts, rather than true biological signals (Hou et al., 2020; Andrews and Martin, 2019). SAVER performs better than MAGIC and ALRA in mitigating noise due to batch effects. However, SAVER recovers a lower resolution of cell type heterogeneity compared with FFAVES, MAGIC, and ALRA, indicated by looser connectivity between local cell populations.

In Figure 5B, we use silhouette scores for six cell type clusters to quantitatively assess imputation performance. Imputation via FFAVES recovers cell clusters that closely resemble the ground truth. The silhouette scores after MAGIC imputation suggest data overfitting: samples appear considerably more similar to each other than in the ground truth. Such overfitting is an example of how metrics such as the silhouette score can be misleading without a ground truth for context. Higher silhouette scores can be incorrectly assumed to be synonymous with better imputation. The ALRA silhouette scores are similar to FFAVES and the ground truth, albeit with some slight overfitting. Finally, the SAVER silhouette scores are worse than the dropout + batch effect synthetic data. In particular, some cells have negative silhouette scores, indicating that they no longer cluster with their original cell type labels.

ESFW accurately discriminates informative features

The ESFW algorithm is designed to weight features using an unsupervised process that identifies combinatorial gene expression patterns. Higher weights indicate those that are more likely to be informative for sample identities/clusters. For a detailed description of ESFW see supplemental information section 5. We used ESFW to calculate the feature weights for our dropout + batch effect synthetic data before/after FFAVES, as well as for the ground truth data. Even without applying FFAVES, a bimodal distribution of weights emerges (Figure 5C, top). Those genes with feature weights close to or equal to zero are the uninformative genes. The second group with feature weights around 0.001 comprise over 97% of the 969 highly structured informative genes. However, as expected, these highly structured genes in the dropout + batch effect synthetic data have feature weights lower than that of the ground truth data. After applying FFAVES, we find that a large proportion of the informative genes have feature weights that closely resemble those of the ground truth (Figure 5C, bottom), further demonstrating that FFAVES accurately re-captures feature dependencies. The small fraction of informative genes that retain feature weights of around 0.001 comprise the same genes previously suggested to have ground truth minority state cardinalities too low for FFAVES to identify and repair.

Since ESFW provides a score for each feature with regard to sample structure, we can form a ranked list of gene importance. We can use ranked gene lists from ESFW and other feature selection software to compare their ability to distinguish between the highly structured cell-type-specific genes and the uninformative genes in our synthetic data. Because feature selection is a common and important step in workflows for analyzing scRNA-seq data, several implementations have been developed. We selected three unsupervised and popular tools (Yip et al., 2018) for comparison: Scran (Lun et al., 2016), Seurat (Hao et al., 2021), and scry (Townes et al., 2019).

(B) Silhouette scores for each of the six main clusters of cells in the synthetic dataset. Black dashed lines mark the silhouette scores of the ground truth data prior to the introduction of FNs.
(C) Feature importance weights for all genes in the synthetic data according to ESFW. Top: feature weights estimated from the synthetic data with FNs introduced to the ground truth. Bottom: feature weights estimates after FFAVES has identified statistically significant divergent data points.
(D) Precision/recall curves for distinguishing structured and randomly expressed genes. Each line is generated from the ranked gene lists of the respective feature selection software.

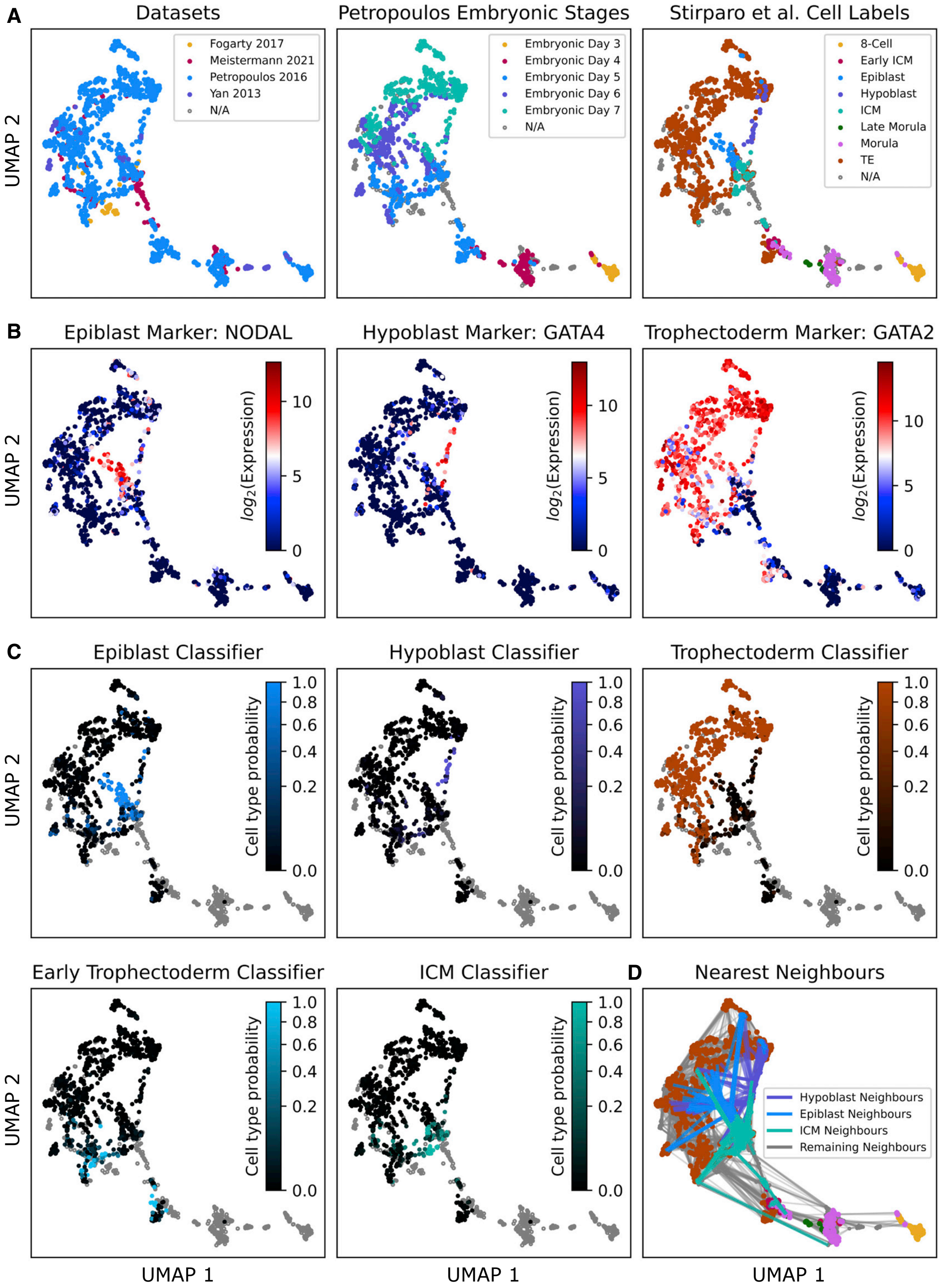

Figure 6. Independent validation of the FFAVES + ESFW human pre-implantation embryo embedding

(A) The FFAVES + ESFW UMAP embedding overlaid with different label information: (left to right) the datasets that samples originate from, the time point labels from the Petropoulos dataset, and the cell type labels for the Petropoulos dataset that were independently assigned by Stirparo et al., (2018).

(legend continued on next page)

Figure 5D presents the precision/recall curves from applying each tool to our synthetic data. For all plots except the results obtained after applying FFAVES to the dropout synthetic data (orange dashed line), feature selection was performed on the synthetic data with dropouts + batch effects. The ESFW precision/recall curves show high discrimination between structured and randomly expressed features, up to a recall of 0.97. Conversely, Seurat, Scran, and scry show a considerable drop in precision at recall values less than 0.1, indicating that these methods struggle to differentiate between the informative and uninformative genes. Precision scores below the red dashed line are those where a higher fraction of uninformative genes are inspected than would on average be observed under random sampling of genes.

FFAVES and ESFW reveal a high-resolution scRNA-seq embedding of the human pre-implantation embryo

To test the utility of ES for examination of real biological systems, we considered human pre-implantation embryo scRNA-seq data containing 1,751 cells and 34,054 genes, compiled by Meistermann et al. (2021). Using FFAVES and ESFW, we identified a set of 3,700 highly informative genes. Restricting the expression matrix to these genes, we generated UMAPs that identify distinct cell type populations along the developmental time course (Figure 6A). Thus, the embryonic day (E) labels progress chronologically from the bottom right to the top left of the UMAP. Importantly, we generated this high-resolution embedding without any augmentation of the original expression matrix: no data transformations, batch correction, smoothing, or imputation. This gives confidence that any cell similarities or gene expression signatures identified are likely to be biologically significant rather than introduced during computational pre-processing.

An initial comparison of our UMAP embedding with the labels identified by the Meistermann et al. (2021) analysis indicates generally good agreement with their supervised analysis (Figure S6A). However, there are discrepancies among proposed epiblast (Epi) and hypoblast (Hyp) populations, and in contrast to Meistermann, our study identifies a distinct early inner cell mass (ICM) population. Identification of the ICM in human embryos has been a subject of debate that has provoked alternative models of early lineage segregation (Weltner and Lanner 2021).

Independent validation of our UMAP embedding

To examine and validate the UMAP embedding, we compare it against previous analyses of human and primate pre-implantation embryos. Stirparo et al. (2018) analyzed the Petropoulos et al. (2016) dataset (which accounts for 85% of the data in Meistermann et al. 2021) and proposed cell type annotations based on known gene expression signatures and unsupervised clustering. Overlaying Stirparo's annotations onto our embedding, we find distinct groups of cells, indicating that the UMAP identifies cell clusters with specific gene expression signatures (Figure 6A). The increased clustering performance when using the 3,700 ES selected genes compared with the 4,484 HVG genes selected by Meistermann et al. (2021) can at least partially be explained by the ES genes further distinguishing cell type clusters (quantified by silhouette scores) for the ground truth cell types proposed by Stirparo et al. (2018) (Figure S7). Thus, our unsupervised feature selection approach is in better agreement with the supervised analysis of Stirparo et al. (2018) than HVG selection. Overlaying the UMAP with gene expression profiles of canonical Epi, Hyp, and trophectoderm (TE) markers (Stirparo et al., 2018; Amrani et al., 2019) shows consistency with the proposed cell labels (Figures 6B and S8).

To demonstrate that the structure in our UMAP is conserved in independent datasets, we created two sets of cell type classifiers from (1) human pre-implantation embryo cells from Yanagida et al. (2021) and (2) cynomolgus monkeys (*Macaca fascicularis*) pre-implantation embryo cells from Nakamura et al. (2016). We used the scANVI machine learning package (Gayoso et al., 2022) to train cell type classifiers on the cell labels allocated to the reference cells from Yanagida et al. or Nakamura et al. We then applied the classifiers to our UMAP embedding to predict cell types based on gene expression signatures. These analyses showed good agreement with the clusters identified in our embedding, with each cell type classifier scoring cells most highly in the expected regions of the UMAP (Figures 6C and S6B).

Together, the cell type labels from Stirparo et al. (2018), the cell-type-specific gene expression profiles, and the cell type classifiers created from independent human or Macaca pre-implantation embryo datasets provide four independent validations of our UMAP embedding. We conclude that FFAVES and ESFW have provided a higher resolution of cell type/gene expression dynamics than achieved by previous analyses of these data.

(B) Example epiblast, hypoblast, and trophectoderm marker expression. See Figure S8 for more examples.
(C) Predicted cell type probabilities of individual cells from a classifier trained on human pre-implantation embryo scRNA-seq data from the independent Yanagida et al., (2021) dataset. Gray samples are those that were not processed by the classifier to avoid confounding variables such as batch effects. See Figure S6B for the same analysis with Macaca classifiers.
(D) Nearest neighbor embedding where each cell is connected by lines to their 10 most similar samples according to gene expression. See Figure S6C for individual cell type nearest neighbor embeddings.

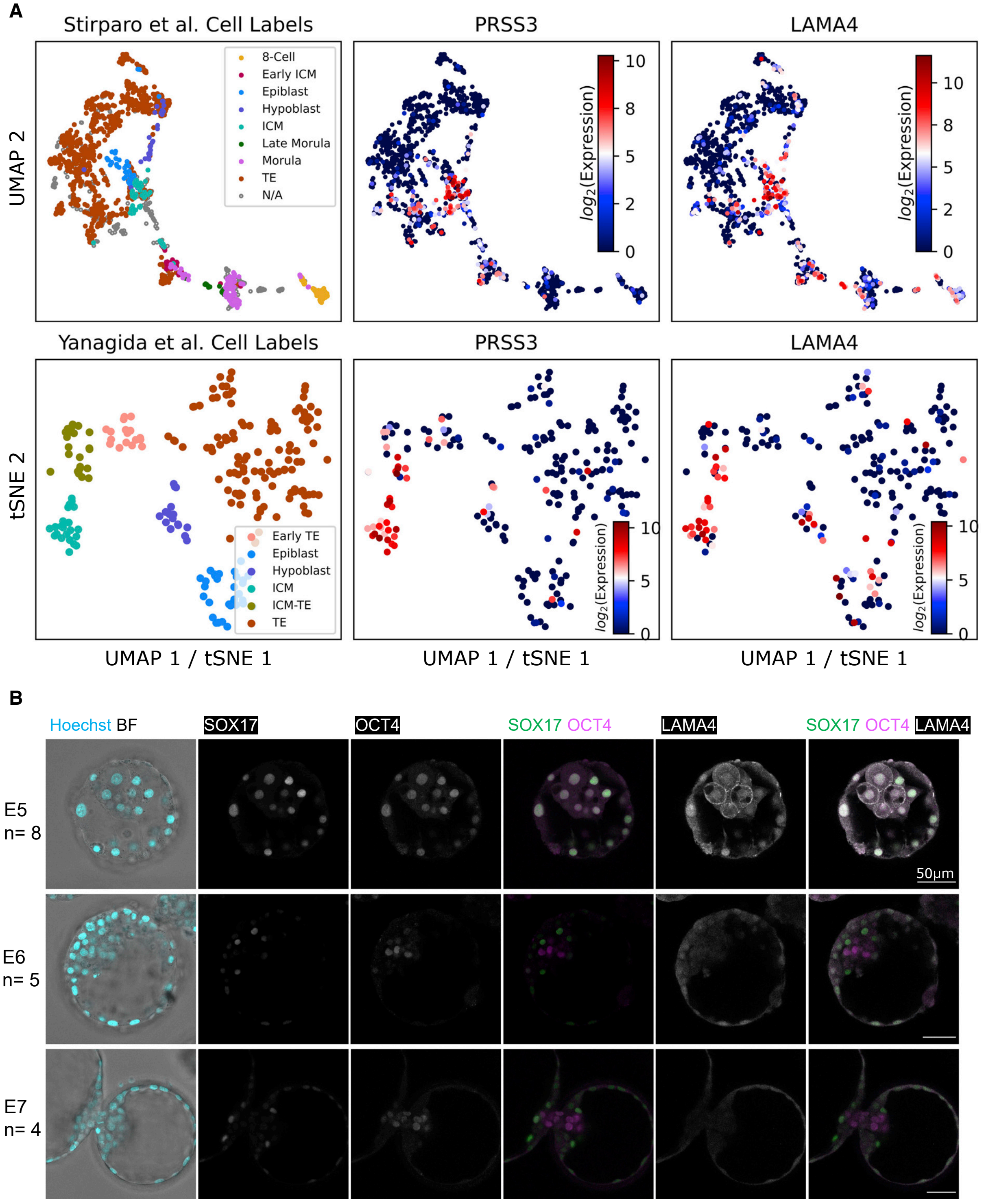

Figure 7. Identification of potential ICM markers

(A) ICM markers were selected based on localized expression in the ICM population of the FFAVES + ESFW UMAP embedding (top row) corroborated in the tSNE embedding generated by Yanagida et al. (bottom row). See Figure S9 and online methods for additional proposed ICM markers.

(legend continued on next page)

Defining the human pre-implantation embryo inner cell mass

There are two prevailing hypotheses regarding the establishment of the Epi, Hyp, and TE lineages during human pre-implantation development. Petropoulos et al. (2016) concluded from scRNA-seq analysis that the three lineages may emerge simultaneously. However, mouse experimental embryology studies have established a two-step model, with the first cell fate decision segregating TE from ICM at the late morula stage (Chazaud and Yamanaka 2016), after which the ICM differentiates into Epi and Hyp in the blastocyst. More recently, Meistermann et al. (2021) analyzed human and mouse pre-implantation embryo scRNA-seq data with an aim to resolve which model is operative. Although Meistermann et al. (2021) found supporting evidence for the two-step model in human development, they were unable to confidently identify an ICM population. In the absence of a clear ICM population, Meistermann et al. (2021) surmised that Hyp cells may emerge from the Epi.

In the FFAVES/ESFW UMAP embedding, divergence into either TE or ICM populations is apparent at E5 (Figures 6A and 6C). Proceeding from E5 to E6/7, the ICM cells differentiate into Epi and Hyp. As mentioned before, the proposed E5 ICM population has been previously suggested by Stirparo et al. (2018) but could not be resolved through dimensionality reduction techniques. The existence of the ICM is further supported by our classifier analysis utilizing independently generated ICM gene expression signatures (Figures 6C and S6B). Furthermore, by nearest neighbor analysis (inspecting the 10 most similar cells based on gene expression for the suggested ICM, Epi, and Hyp cells), we find that the Epi and Hyp cells are each connected to the ICM population, but they have very little connectivity to each other (Figures 6D and S6C). The lack of connectivity between the Epi and Hyp cells supports the hypothesis that both differentiate from the ICM, rather than Hyp emerging from Epi.

Identification of a distinct ICM population enables us to suggest gene markers for future studies. We sought to identify genes whose expression was localized to the human ICM cells in our UMAP embedding. In our GitHub repository (see experimental procedures), we describe how these markers were identified and list more potential ICM markers. For validation, we examined their expression in an independent tSNE embedding from Yanagida et al. (2021) (Figures 7A and S9). We present two broad types of ICM markers. Those such as *FGF1* and *PRSS3* display expression specifically in the ICM-labeled cells in both embeddings. The second set exhibit upregulated expression at E4 in addition to E5 ICM, and they are markedly downregulated in the Epi, Hyp, and TE populations. Examples of this group are *BHMT* and *SPIC*. We note that previous studies have proposed *SPIC* and *PRSS3* as human ICM markers (Singh et al., 2019).

Immunostaining validates LAMA4 as an ICM marker that is extinguished in epiblast

To test whether markers identified from our UMAP embedding localize to the ICM, we performed immunostaining of LAMA4 on embryonic days 5–7 (E5–E7) human embryos. We selected LAMA4 as a cell surface protein with relatively high expression levels ($>log_2 10$) within the ICM cells (Figure 7A) and with an available antibody reagent. At E5 (mid-blastocyst), LAMA4 is clearly localized to the cell surface of the ICM cells (Figure 7B). Co-expression of OCT4 and SOX17 confirms that hypoblast and epiblast lineages are not yet specified (Niakan and Eggan 2013). By E6/7 (late blastocyst), LAMA4 expression is downregulated, while OCT4+ and SOX17+ cells are segregated to epiblast and hypoblast fates respectively. These results provide validation that our embedding can identify the ICM population and specific markers prior to epiblast and hypoblast specification.

DISCUSSION

Over the last decade, the advancement of next-generation sequencing (NGS) techniques has markedly increased the types and quantity of data that can be obtained on genome control of cell behavior (Anaparthy et al., 2019). While this increase in molecular information is exciting, it also presents new challenges around how best to analyze large, high-dimensional datasets to generate biological insight (Angerer et al., 2017). In this work we present entropy sorting, a mathematical framework that quantifies the correlations between features (genes) in a high dimensional dataset as a sorting problem. The theory of ES is encoded in two algorithms: FFAVES and ESFW. Together, these provide unsupervised pre-processing to increase the resolution of information extracted from scRNA-seq data, and high-dimensional data in general.

To demonstrate the effectiveness of ES, we applied our software to both synthetic and experimental scRNA-seq datasets. On synthetic data with known ground truth, we demonstrate that FFAVES and ESFW perform markedly better than popular HVG identification software at discriminating highly correlated and randomly expressed genes. When compared with other popular imputation software

(B) Confocal images of human embryos immunostained for LAMA4 cell surface protein together with SOX17 and OCT4 nuclear transcription factors. Nuclei are visualized with Hoechst staining. The zona pellucida has been removed at E5 and E6 but not at E7 due to the embryo beginning to hatch. Staining patterns were consistent for all embryos examined: E5, n = 8; E6, n = 5; E7, n = 4. Scale bar represents 50 μm.

we show that FFAVES can identify FNs and FPs with high accuracy, and it facilitates imputation such that ground truth cell-cell similarities are recovered. Furthermore, ESFW was shown to outperform current popular methods in performing feature selection to distinguish cell-type-specific genes from randomly expressed genes.

Applied to scRNA-seq data from human pre-implantation embryos (Meistermann et al., 2021), FFAVES and ESFW identified a subset of 3,700 genes that were highly predictive of cell state. Filtering to these highly structured genes yielded UMAP embeddings with a higher resolution of gene expression dynamics during pre-implantation development than previously observed. Crucially, this was achieved by unsupervised filtering, without changing any values in the original gene expression matrix. Notably, FFAVES/ESFW revealed a distinct ICM population that precedes both the epiblast and hypoblast lineages. These analyses provide evidence for the two-step model of pre-implantation lineage segregation, which is well established in mouse development but has been disputed in human embryos due to failure of previous analyses to discriminate a distinct ICM population (Petropoulos et al., 2016; Meistermann et al., 2021; Stirparo et al., 2018). Immunostaining for LAMA4 shows ICM-specific expression at E5 with downregulation in epiblast and hypoblast at E6/E7. This result substantiates the reliability of our embedding and demonstrates the potential to identify new lineage-specific markers for analysis of early human development.

FFAVES and ESFW should be viewed as pre-processing steps that help to maximize the signal of highly structured features. We chose not to apply any batch correction, imputation, or feature extraction methods (other than UMAPs for visualization) to the human embryo data. In doing so we show that ES is able to address the CoD in a manner that elucidates hidden structure in scRNA-seq data simply by removing spurious/uninformative features, rather than needing to augment or smooth the data. However, it may be possible to gain an even higher resolution of gene expression dynamics by applying other scRNA-seq analysis tools (Wu and Zhang 2020), after application of FFAVES/ESFW.

ES has the potential to be useful in other domains. Within the scope of NGS techniques, ES should be straightforward to apply to methods such as single-cell Assay for Transposase-Accessible Chromatin sequencing (ATAC-seq) and Bisulfite sequencing (BS-seq). Furthermore, the requirement to provide ES with discrete data may be advantageous for multi-omics single-cell analyses (Anaparthy et al., 2019), which provide simultaneous readouts for multiple NGS techniques. Each technique can produce very different types of numerical outputs, so combining them into a single dataset/readout is non-trivial. ES has the potential to overcome this challenge by discretizing the data and framing the problem as the identification of functional relationships between the presence/absence of mRNA, chromatin accessibility/inaccessibility, or sequence methylation/non-methylation. Therefore, ES offers the possibility of combining different types of data for coherent analysis. Finally, beyond NGS techniques, ES should be a powerful tool for reducing the complexity of a wide variety of high-dimensional datasets, such as medical diagnostic or marketing data, in an efficient and unsupervised manner.

EXPERIMENTAL PROCEDURES

Human embryos

Supernumerary frozen human embryos were donated with informed consent by couples undergoing *in vitro* fertility treatment. Use of human embryos in this research is approved by the Multi-Centre Research Ethics Committee, approval O4/MRE03/44, and licensed by the Human Embryology Fertilization Authority of the United Kingdom, research license R0178.

Detailed descriptions of embryo preparation and embryo staining experimental procedures can be found in the supplemental experimental procedures.

DATA AND CODE AVAILABILITY

Computational workflows and data used for the generation of results in this article are available through the following sources.

The human pre-implantation embryo data may be found in a permanent Mendeley Data repository (Mendely Data: https://doi.org/10.17632/689pm8s7jc.1). This repository also contains detailed workflows to reproduce our results.

The data used to create our UMAP embedding are a combination of raw counts scRNA-seq data from Yan et al. (2013), Petropoulos et al. (2016), Fogarty et al. (2017), and Meistermann et al. (2021), which were compiled into a single gene expression matrix kindly provided by Meistermann et al. (2021). For information regarding data processing, please refer to their manuscript.

The Yanagida et al. (2021) human embryo data analyzed in this paper are available via GEO accession number GSE171820. The tSNE used in this paper for visualizing the data was kindly provided by the authors.

The Nakamura et al. (2016) Macaca embryo data analyzed in this paper are available via GEO accession number GSE74767.

Instructions to install FFAVES and ESFW can be found at https://github.com/aradley/FFAVES. This repository also contains the synthetic data and workflows to reproduce the synthetic data results in this article.

SUPPLEMENTAL INFORMATION

Supplemental information can be found online at https://doi.org/10.1016/j.stemcr.2022.09.007.

AUTHOR CONTRIBUTIONS

Conceptualization: A.R., S-J.D., and A.S.; data curation: A.R., E.C-S., and J.N.; formal analysis: A.R.; funding acquisition: S-J.D. and A.S.; investigation: A.R., E.C-S., and J.N.; methodology: A.R.; project administration: S-J.D. and A.S.; resources: S-J.D., A.S., and

J.N.; software: A.R.; supervision: S-J.D. and A.S.; validation: A.R., E.C-S., and J.N.; visualization: A.R. and E.C-S.; writing – original draft: A.R.; writing – review & editing: S-J.D. and A.S.

ACKNOWLEDGMENTS

This research was supported by the Biotechnology and Biological Sciences Research Council (BBSRC, grant number BB/P021573/1). A.R. was funded by a BBSRC PhD studentship (1943266) with co-funding from the Microsoft Research PhD scholarship program. J.N. and E.C.-S. were funded by the BBSRC grant number BB/T007044/2. A.S. is a Medical Research Council Professor (G1100526/1). We are grateful to Lawrence Bates for assistance with human embryo thawing.

CONFLICT OF INTERESTS

Sara-Jane Dunn was an employee at Microsoft Research during this study and is currently employed at DeepMind. Microsoft Research provided co-funding for Arthur Radley's research council studentship and access to computational resources. Neither Microsoft Research nor DeepMind have directed any aspect of the study nor exerted any commercial rights over the results.

Received: April 8, 2022
Revised: September 15, 2022
Accepted: September 16, 2022
Published: October 13, 2022

REFERENCES

Altman, N., and Krzywinski, M. (2018). The curse(s) of dimensionality*15*, 399–400. https://doi.org/10.1038/s41592-018- 0019-x.

Amrani, K.E., Alanis-Lobato, G., Mah, N., Kurtz, A., and Andrade-Navarro, M.A. (2019). Detection of condition-specific marker genes from RNA-seq data with MGFR. PeerJ *7*, e6970. https://doi.org/10.7717/peerj.6970.

Anaparthy, N., Ho, Y.J., Martelotto, L., Hammell, M., and Hicks, J. (2019). Single-cell applications of next-generation sequencing. Cold Spring Harb. Perspect. Med. *9*. https://doi.org/10.1101/CSHPERSPECT.A026898.

Andrews, T.S., and Martin, H. (2019). False signals induced by single-cell imputation. F1000Res., 1740. https://doi.org/10.12688/f1000research.16613.2.

Angerer, P., Simon, L., Tritschler, S., Wolf, F.A., Fischer, D., and Theis, F.J. (2017). Single cells make big data: new challenges and opportunities in transcriptomics. Curr. Opin. Syst. Biol. *4*, 85–91. https://doi.org/10.1016/J.COISB.2017.07.004.

Bellman, R. (1967). Dynamic programming. Math. Sci. Eng. *40*, 101–137. https://doi.org/10.1016/S0076-5392(08)61063-2.

Cannoodt, R., Saelens, W., Deconinck, L., and Saeys, Y. (2021). Spearheading future omics analyses using dyngen, a multi-modal simulator of single cells. Nat. Commun. *12*, 1–9. https://doi.org/10.1038/s41467-021-24152-2.

Chazaud, C., and Yamanaka, Y. (2016). Lineage specification in the mouse preimplantation embryo. Development *143*, 1063–1074. https://doi.org/10.1242/dev.128314.

van Dijk, D., Sharma, R., Nainys, J., Yim, K., Kathail, P., Carr, A.J., Burdziak, C., Moon, K.R., Chaffer, C.L., Pattabiraman, D., et al. (2018). Recovering gene interactions from single-cell data using data diffusion. Cell *174*, 716–729.e27. https://doi.org/10.1016/j.cell.2018.05.061.

Fogarty, N.M.E., McCarthy, A., Snijders, K.E., Powell, B.E., Kubikova, N., Blakeley, P., Lea, R., Elder, K., Wamaitha, S.E., Kim, D., et al. (2017). Genome editing reveals a role for OCT4 in human embryogenesis. Nature *550*, 67–73. https://doi.org/10.1038/nature24033.

Gayoso, A., Lopez, R., Xing, G., Boyeau, P., Valiollah Pour Amiri, V., Hong, J., Wu, K., Jayasuriya, M., Mehlman, E., Langevin, M., et al. (2022). A Python library for probabilistic analysis of single-cell omics data. Nat. Biotechnol. *40*, 163–166. https://doi.org/10.1038/s41587- 021-01206-w.

Hao, Y., Hao, S., Andersen-Nissen, E., Mauck, W.M., 3rd, Zheng, S., Butler, A., Lee, M.J., Wilk, A.J., Darby, C., Zager, M., et al. (2021). Integrated analysis of multimodal single-cell data. Cell *184*, 3573–3587.e29. https://doi.org/10.1016/j.cell.2021.04.048.

Hou, W., Ji, Z., Ji, H., and Hicks, S.C. (2020). A systematic evaluation of single-cell RNA-sequencing imputation methods. Genome Biol. *21*, 218. https://doi.org/10.1186/s13059-020-02132-x.

Huang, M., Wang, J., Torre, E., Dueck, H., Shaffer, S., Bonasio, R., Murray, J.I., Raj, A., Li, M., and Zhang, N.R. (2018). SAVER: gene expression recovery for single-cell RNA sequencing. Nat. Methods *15*, 539–542. https://doi.org/10.1038/s41592-018-0033-z.

Källberg, D., Vidman, L., and Rydén, P. (2021). Comparison of methods for feature selection in clustering of high-dimensional RNA-sequencing data to identify cancer subtypes. Front. Genet. *12*, 217. https://doi.org/10.3389/fgene.2021.632620.

Kiselev, V.Y., Andrews, T.S., and Hemberg, M. (2019). Challenges in unsupervised clustering of single-cell RNA-seq data. Nat. Rev. Genet. *20*, 273–282. https://doi.org/10.1038/s41576-018-0088-9.

Linderman, G.C., Zhao, J., Roulis, M., Bielecki, P., Flavell, R.A., Nadler, B., and Kluger, Y. (2022). Zero-preserving imputation of single-cell RNA-seq data. Nat. Commun. *13*. https://doi.org/10.1038/s41467-021-27729-z.

Lun, A.T.L., McCarthy, D.J., and Marioni, J.C. (2016). A step-by-step workflow for low-level analysis of single- cell RNA-seq data with Bioconductor. F1000Res. *5*. https://doi.org/10.12688/F1000RESEARCH.9501.1.

McInnes, L., Healy, J., Saul, N., and GroBberger, L. (2018). UMAP: Uniform Manifold approximation and projection. J. Open Source Softw. *3.29*, 861. https://doi.org/10.21105/JOSS.00861.

Meistermann, D., Bruneau, A., Loubersac, S., Reignier, A., Firmin, J., Francois-Campion, V., Kilens, S., Lelievre, Y., Lammers, J., Feyeux, M., et al. (2021). Integrated pseudotime analysis of human pre-implantation embryo single-cell transcrip- tomes reveals the dynamics of lineage specification. Cell Stem Cell *28*, 1625–1640.e6. https://doi.org/10.1016/j.stem. 2021.04.027.

Nakamura, T., Okamoto, I., Sasaki, K., Yabuta, Y., Iwatani, C., Tsuchiya, H., Seita, Y., Nakamura, S., Yamamoto, T., and Saitou, M. (2016). A developmental coordinate of pluripotency among mice, monkeys and humans. Nature. https://doi.org/10.1038/nature19096.

Niakan, K.K., and Eggan, K. (2013). Analysis of human embryos from zygote to blastocyst reveals distinct geneexpression patterns relative to the mouse. Dev. Biol. *375*, 54–64. https://doi.org/10.1016/j.ydbio. 2012.12.008.

Petropoulos, S., Edsgard, D., Reinius, B., Deng, Q., Panula, S., Codeluppi, S., Plaza Reyes, A., Linnarsson, S., Sandberg, R., and Lanner, F. (2016). Single-cell RNA-seq reveals lineage and X chromosome dynamics in human preimplan- tation embryos. Cell *165*, 1012–1026. https://doi.org/10.1016/J.CELL.2016.03.023.

Quinlan, J.R. (1986). Induction of decision trees. Mach. Learn. *1*, 81–106. https://doi.org/10.1007/bf00116251.

Ramskö Ld, D., Wang, E.T., Burge, C.B., and Sandberg, R. (2009). An abundance of ubiquitously expressed genes revealed by tissue transcriptome sequence data. PLoS Comput. Biol. *5*, 1000598. https://doi.org/10.1371/journal.pcbi.1000598.

Singh, M., Widmann, T.J., Bansal, V., Cortes, J.L., Schumann, G.G., Wunderlich, S., Martin, U., Garcia-Canadas, M., Garcia-Perez, J.L., Hurst, L.D., and Izsvák, Z. (2019). The selection arena in early human blastocysts resolves the pluripotent inner cell mass. Preprint at bioRxiv, 318329. https://doi.org/10.1101/318329.

Stirparo, G.G., Boroviak, T., Guo, G., Nichols, J., Smith, A., and Bertone, P. (2018). Integrated analysis of single-cell embryo data yields a unified transcriptome signature for the human pre-implantation epiblast. Development *145*. https://doi.org/10.1242/dev.158501.

Tang, F., Barbacioru, C., Wang, Y., Nordman, E., Lee, C., Xu, N., Wang, X., Bodeau, J., Tuch, B.B., Siddiqui, A., et al. (2009). mRNA-Seq whole-transcriptome analysis of a single cell. Nat. Methods *6*, 377–382. https://doi.org/10.1038/nmeth.1315.

Townes, F.W., Hicks, S.C., Aryee, M.J., and Irizarry, R.A. (2019). Feature selection and dimension reduction for single-cell RNA-Seq based on a multinomial model. Genome Biol. *20*, 295. https://doi.org/10.1186/S13059-019-1861-6.

Weltner, J., and Lanner, F. (2021). Refined transcriptional blueprint of human preimplantation embryos. Cell Stem Cell *28*, 1503–1504. https://doi.org/10.1016/J.STEM.2021.08.011.

Wu, Y., and Zhang, K. (2020). Tools for the analysis of high-dimensional single-cell RNA sequencing data. Nat. Rev. Nephrol. https://doi.org/10.1038/s41581-020-0262-0.

Saeys, Y., Inza, I., and Larrañaga, P. (2007). A review of feature selection techniques in bioinformatics. Bioinformatics *23*, 2507–2517. https://doi.org/10.1093/BIOINFORMATICS/BTM344.

Yan, L., Yang, M., Guo, H., Yang, L., Wu, J., Li, R., Liu, P., Lian, Y., Zheng, X., Yan, J., et al. (2013). Single-cell RNA-Seq profiling of human preimplantation embryos and embryonic stem cells. Nat. Struct. Mol. Biol. *20*, 1131–1139. https://doi.org/10.1038/nsmb.2660.

Yanagida, A., Spindlow, D., Nichols, J., Dattani, A., Smith, A., and Guo, G. (2021). Naive stem cell blastocyst model captures human embryo lineage segregation. Cell Stem Cell *28*, 1016–1022.e4. https://doi.org/10.1016/j.stem.2021.04.031.

Yip, S.H., Sham, P.C., and Wang, J. (2018). Evaluation of tools for highly variable gene discovery from single-cell RNA-seq data. Brief. Bioinform. *20*, 1583–1589. https://doi.org/10.1093/bib/bby011.

Zimek, A., Schubert, E., and Kriegel, H.P. (2012). A survey on unsupervised outlier detection in high-dimensional numerical data. Stat. Anal. Data Min. *5*, 363–387. https://doi.org/10.1002/sam.11161.

Stem Cell Reports, Volume *18*

Supplemental Information

Entropy sorting of single-cell RNA sequencing data reveals the inner cell mass in the human pre-implantation embryo

Arthur Radley, Elena Corujo-Simon, Jennifer Nichols, Austin Smith, and Sara-Jane Dunn

SUPPLEMENTARY MATERIAL

Supplemental Figures

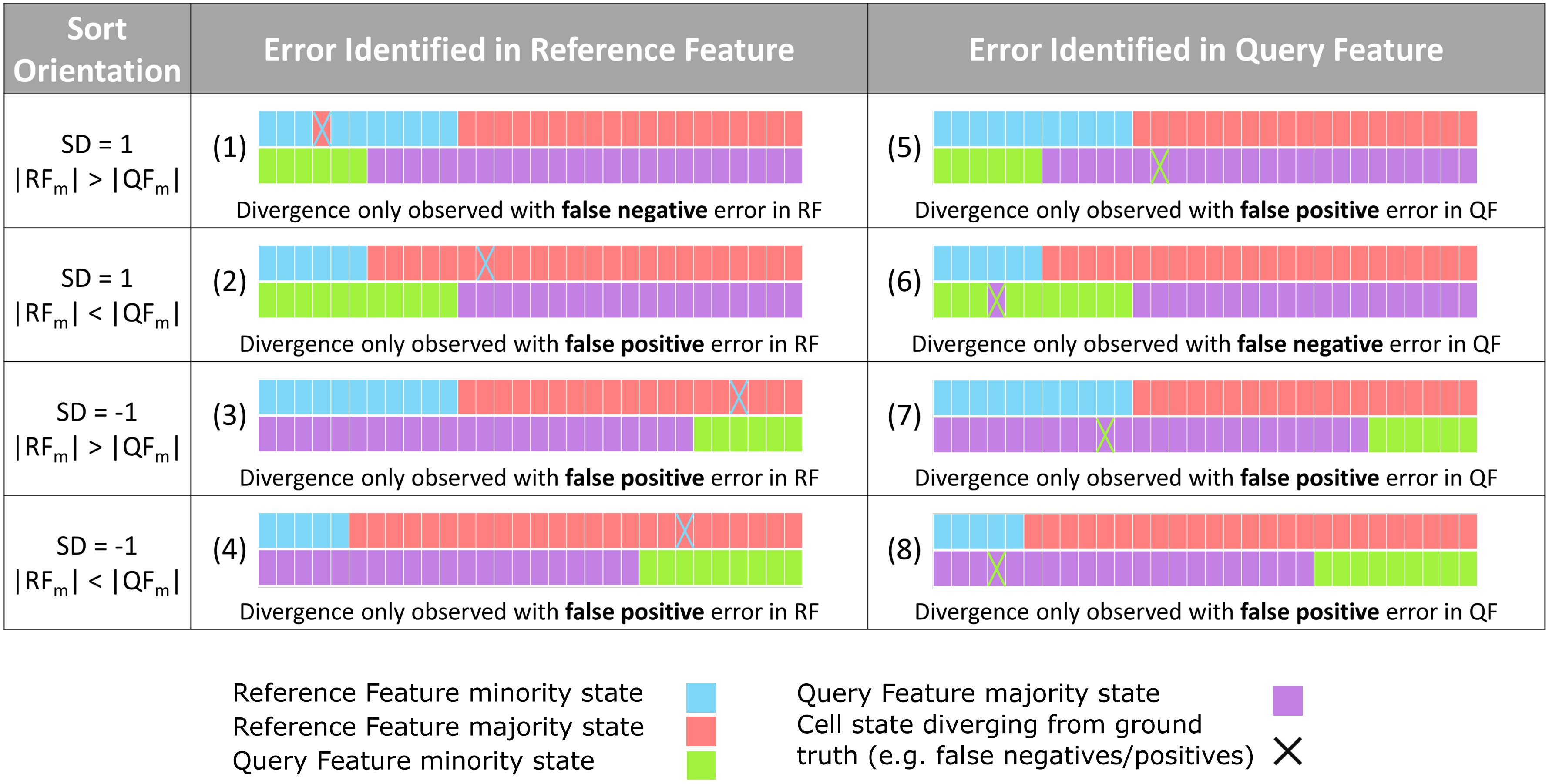

Figure S1. Observable Divergence Error Scenarios. Here we present the eight scenarios in which the introduction of error would lead to observable divergence on an ESE parabola. These scenarios are initially separable by whether the system has a SD of 1 or -1, and whether the number of QF minority states ($|QF_m|$) is greater than or less than the number of RF minority states ($|RF_m|$), as shown in the sort orientation column. These scenarios are subsequently defined by whether the error occurs in the RF or QF. For each scenario we show a basic example where the error would lead to observable divergence.

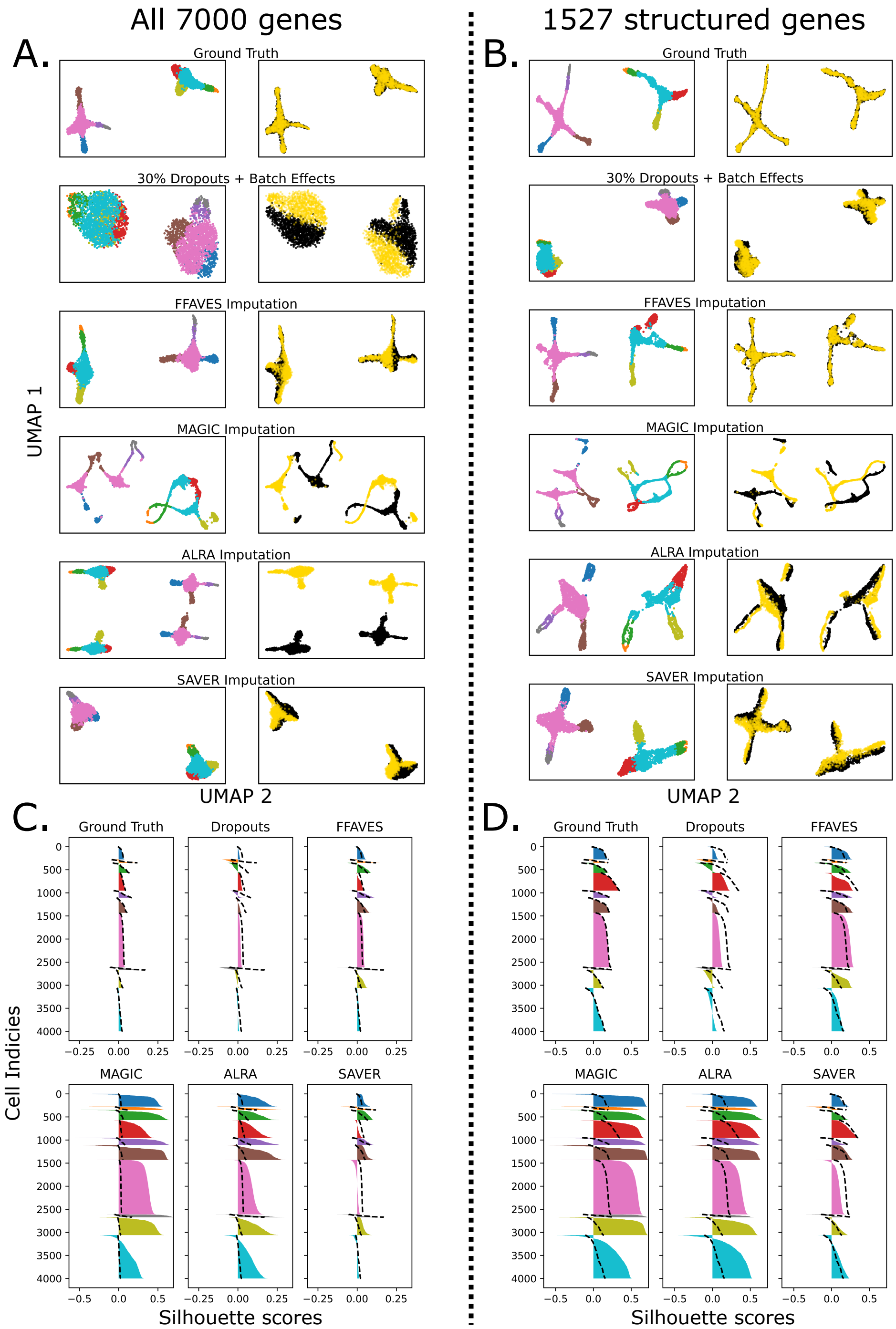

Figure S2. Imputation comparisons on Dyngen simulated data containing 4000 cells and 7000 genes. Related to Fig 5. We simulated a single cell RNA sequencing dataset with 4000 cells and 7000 genes using the Dyngen simulation software (Cannoodt et al. 2021). Of the 7000 genes, 1527 were highly structured genes part of the gene regulatory networks used to simulate cell types. 2878 of the 7000 genes were simulated as part of the house keeping gene regulatory networks and the final 2595 were genes ubiquitously randomly expressed throughout the cells. For the workflow used to create this dataset, see our online data repository. UMAPs of the synthetic data before and after imputation show that FFAVES facilitated imputation performs favourably compared to MAGIC, ALRA and SAVER when considering all 7000 genes **A.**, and just the 1527 structured genes **B.** Left hand columns of the UMAPs are coloured by 10 clusters of cells identified through K-means clustering. Right hand columns show the two batches created through batch specific simulated dropouts. Silhouette plots for 10 distinct clusters of cells, quantify how well the cells cluster together after imputation, compared to the ground truth dataset for all 7000 genes **C.** and just the 1527 structured genes **D.** Dashed lines in the silhouette plots trace the ground truth silhouette scores.

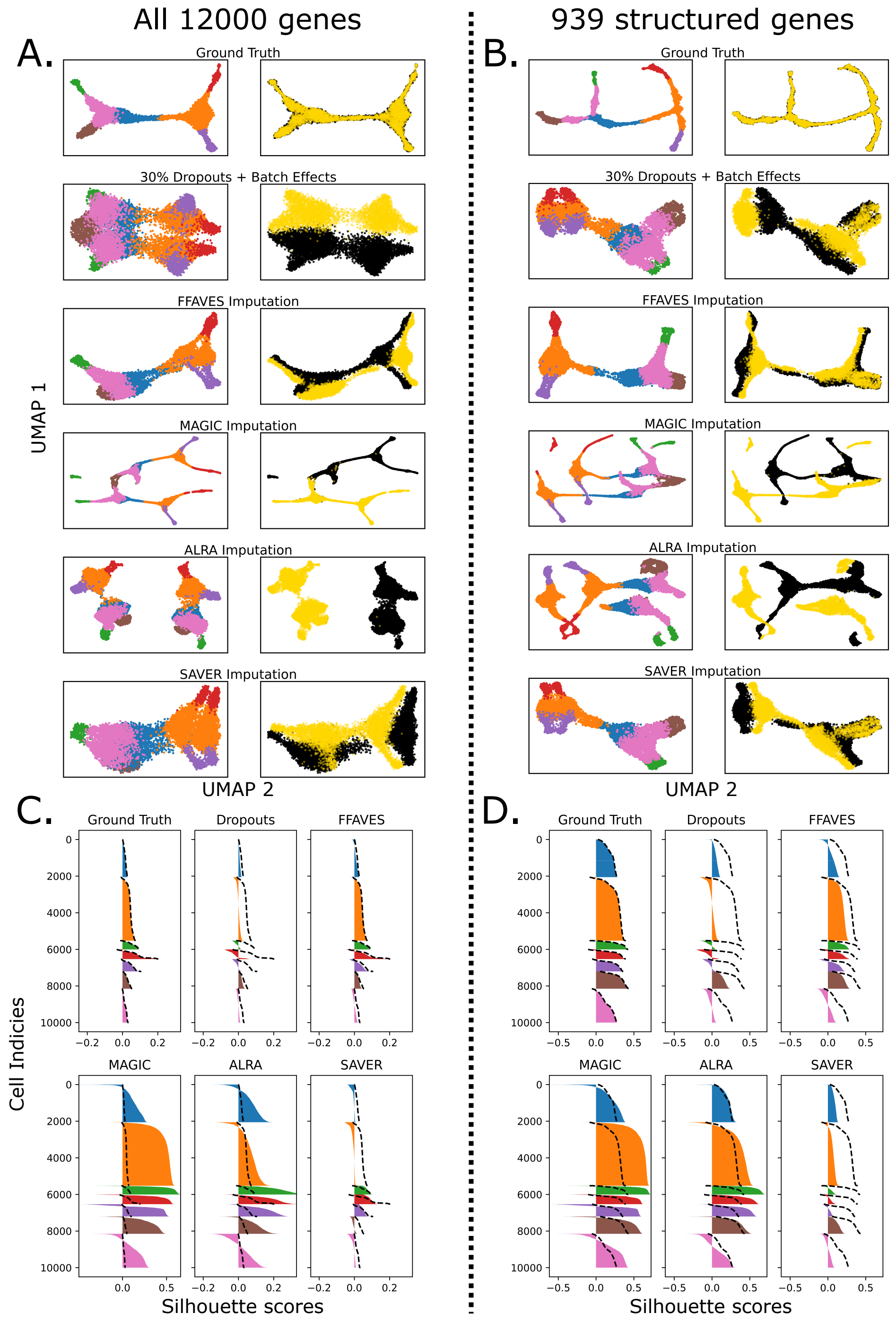

Figure S3. Imputation comparisons on Dyngen simulated data containing 10000 cells and 12000 genes. Related to Fig 5. We simulated a single cell RNA sequencing dataset with 10000 cells and 12000 genes using the Dyngen simulation software (Cannoodt et al. 2021). Of the 12000 genes, 996 were highly structured genes part of the gene regulatory networks used to simulate cell types. 4975 of the 12000 genes were simulated as part of the house keeping gene regulatory networks and the final 6029 were genes ubiquitously randomly expressed throughout the cells. For the workflow used to create this dataset, see our online data repository. UMAPs of the synthetic data before and after imputation show that FFAVES facilitated imputation performs favourably compared to MAGIC, ALRA and SAVER when considering all 12000 genes **A.**, and just the 996 structured genes **B.** Left hand columns of the UMAPs are coloured by 7 clusters of cells identified through K-means clustering. Right hand columns show the two batches created through batch specific simulated dropouts. Silhouette plots for 7 distinct clusters of cells, quantify how well the cells cluster together after imputation, compared to the ground truth dataset for all 12000 genes **C.** and just the 996 structured genes **D.** Dashed lines in the silhouette plots trace the ground truth silhouette scores.

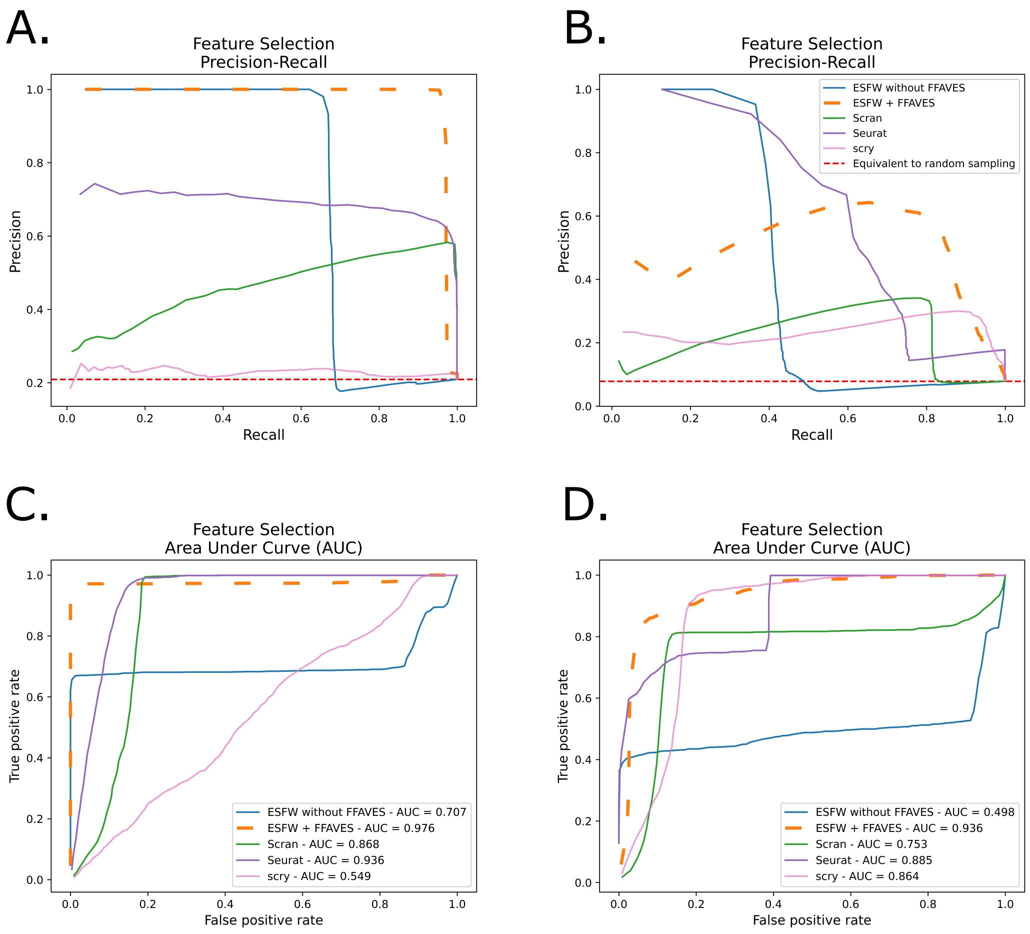

Figure S4. Comparison of feature selection methods on Dyngen simulated datasets. Related to Fig 5. Feature selection using FFAVES + ESFW was compared against Scran, Seurat and scry HVG feature selection software, when applied to the Dyngen synthetic scRNA-seq datasets presented in figures S2 and S3. The precision/recall curves are presented for these datasets in **A.** and **B.** respectively. We note that for our second Dyngen simulated dataset, ESFW without FFAVES does not outperform Seurat in terms of feature selection. ESFW + FFAVES initially appears to perform poorly. This is due to a small number of housekeeping genes having their signal falsely amplified by FFAVES. Once FFAVES + ESFW reaches a recall value of around 0.8 is it enriching highly structured genes considerably more than all other methods. However, to further emphasise that ESFW + FFAVES outperforms the other feature selection methods, we also provide the AUC curves to quantify feature selection performance in **C.** for the Dyngen simulated data in Fig. S2 and **D.** for the the data in Fig. S3. In AUC curves, a higher AUC indicates better performance. Generally AUC curves are not used when there is a significant class in balance (Davis and Goadrich n.d.). Despite there being a high degree of class imbalance in the Dyngen simulated data, since there are far more uninformative genes than structured genes, the AUC curves are useful for further validating the ESFW + FFAVES performance.

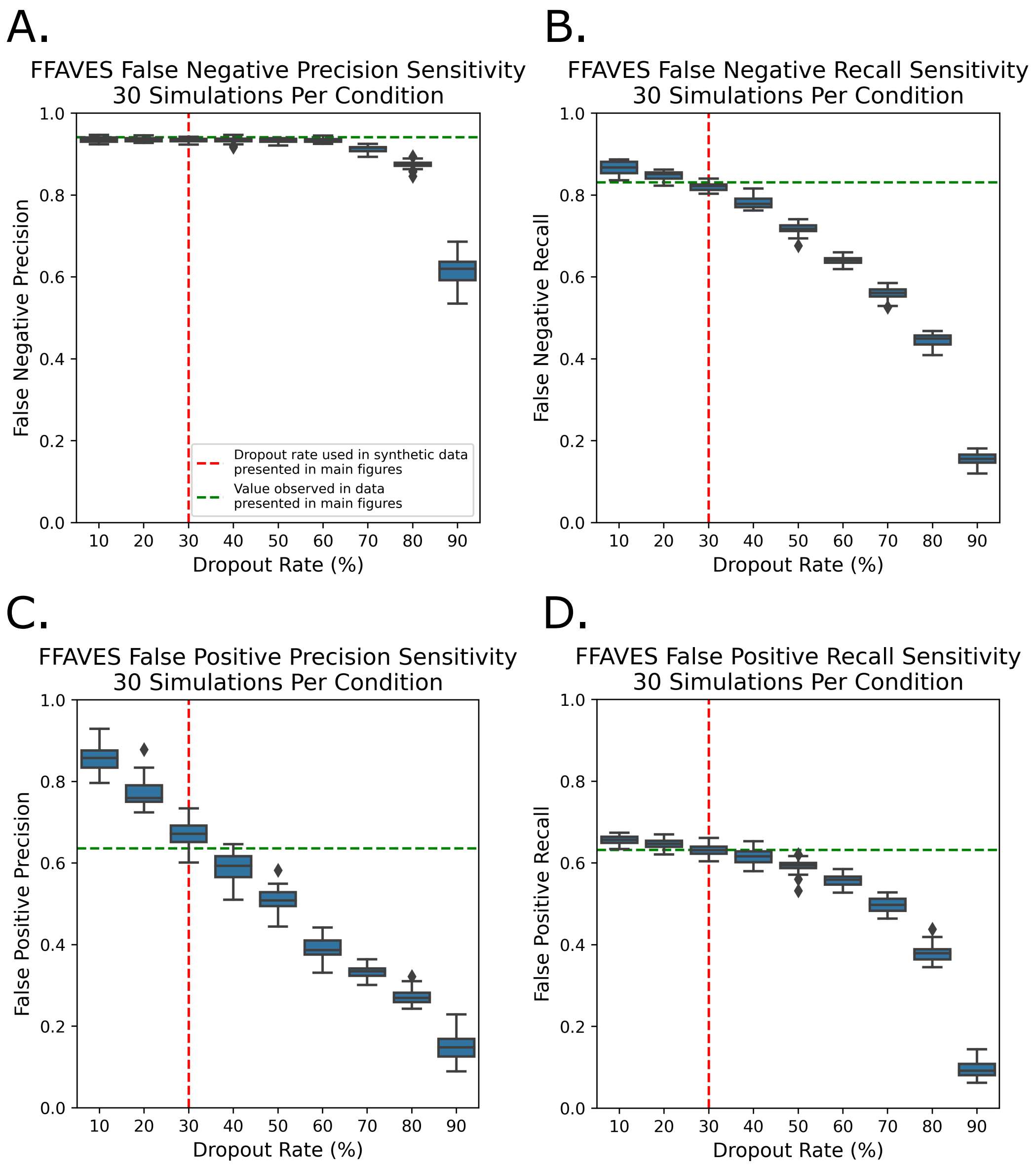

Figure S5. False negative and false positive precision and recall scores are robust to stochastically generated datasets across varying dropout rates. Related to Fig. 4C, D. To verify that FFAVES is robust to stochastically generated synthetic data, we created 30 new datasets in the same manner as was done for the synthetic data presented in the main text (SI 7) and calculate the precision/recall scores for the FPs/FNs identified by FFAVES. This process was repeated while varying the intentionally added dropout rates from 0.1-0.9. The results for each dropout rate are summarised in box plots. Diamond points are data points outside of the interquartile range range. Red dashed lines indicate the dropout rate that was used to generate the synthetic data presented in the main text (30%). Green dashed lines show the scores that were calculated in the data presented in the main text for, **A.** FN precision, **B.** FN recall, **C.** FP precision, and **D.** FP recall.

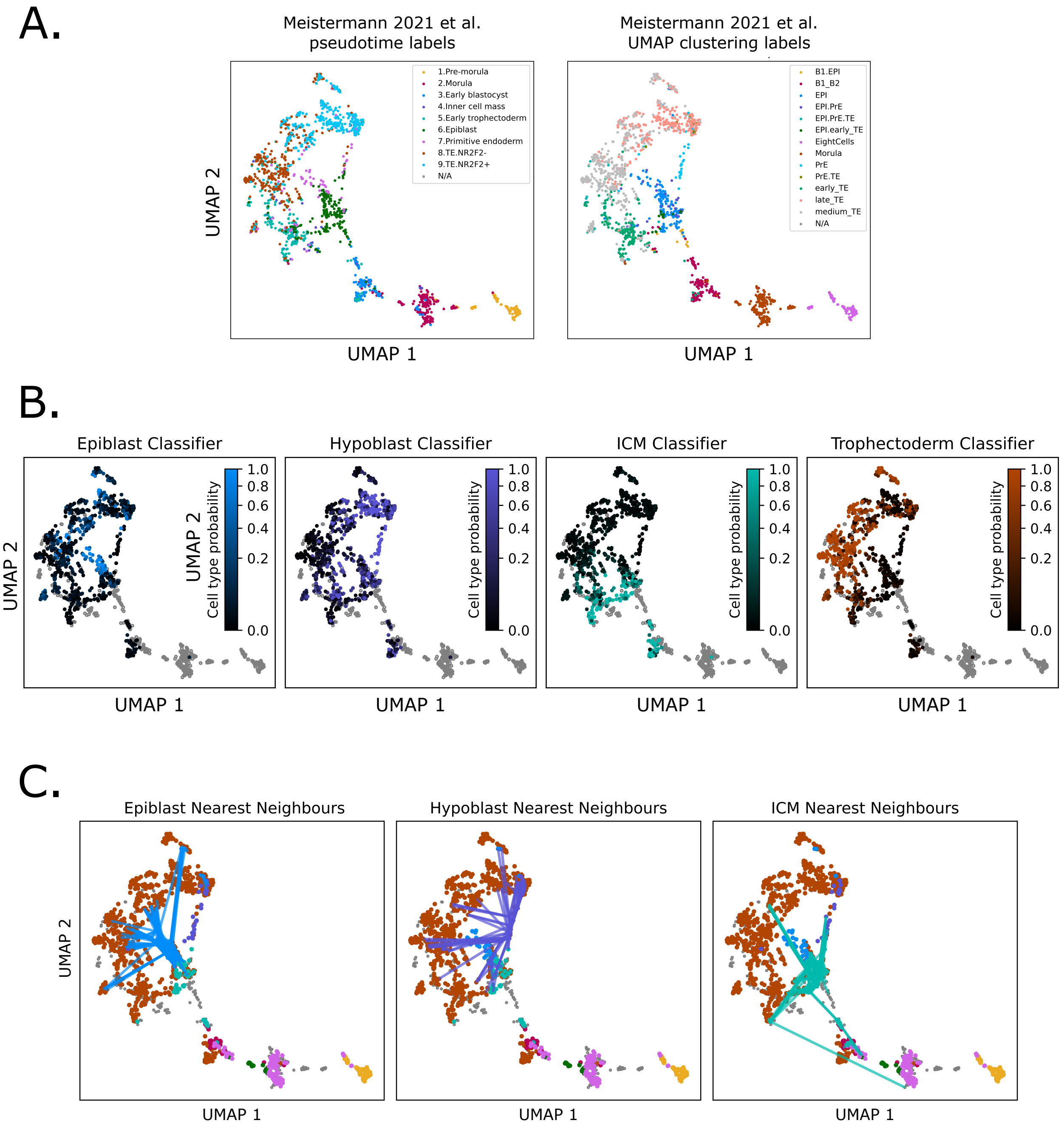

Figure S6. Additional validation of UMAP embedding. Related to Fig 6. A. In their analysis, Meistermann et al. 2021 note that they were unable to isolate a distinct ICM population. By overlaying their assigned labels from their pseudotime analysis (left panel) and their UMAP clustering analysis (right panel), we find that our unsupervised analysis of the scRNA-seq data largely agree with their supervised analysis. However our unsupervised UMAP suggests that in their pseudotime analysis, the epiblast has been incorrectly labeled as the hypoblast (primitive endoderm) and their suggest epiblast population is our suggested inner cell mass cluster. Likewise in their UMAP clustering, their analysis appears to have been unable to separate our proposed inner cell mass cells from the epiblast cells. **B.** Predicted cell type probabilities of individual cells when a classifier trained on Macaca primate pre-implantation embryo scRNA-seq data from the independent Nakamura et al. dataset. Related to Fig. 6C. Grey samples are those that were not processed by the classifier to avoid confounding variables such as batch effects. **C.** Separate nearest neighbour embeddings for epiblast, hypoblast and ICM cells indicate that the epiblast and hypoblast gene expression signatures are more similar to the ICM than to each other. Related to Fig. 6D. Each of the epiblast, hypoblast and ICM cells identified by Stirparo et al. is connected by lines to their 10 most similar samples according to gene expression.

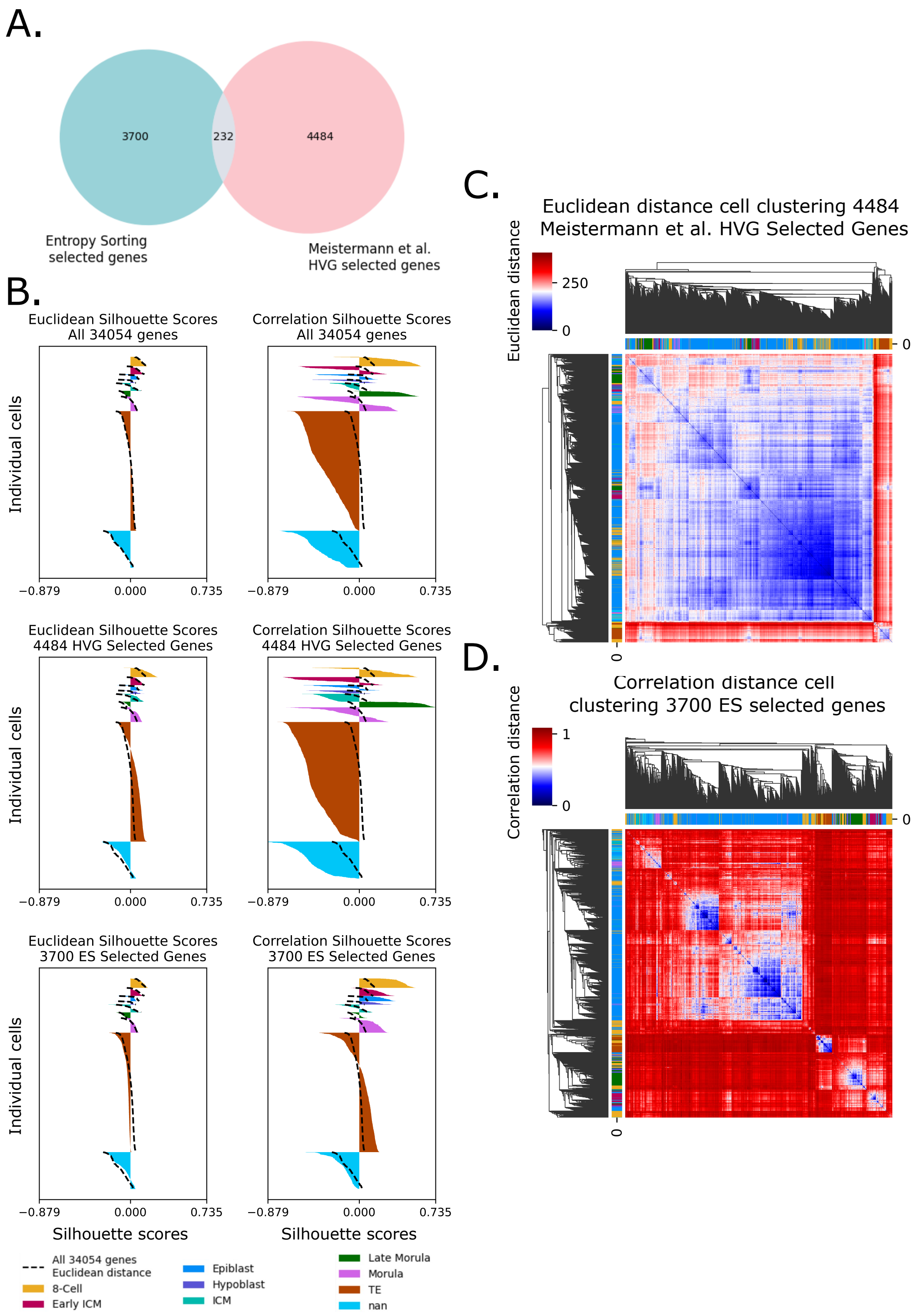

Figure S7. **FFAVES + ESFW selected genes produce a higher resolution of specific cell type clusters than HVG selection in the human pre-implantation embryo data. A.** Overlap of genes selected as informative for cell identity in the Meistermann et al. 2021 human pre-implantation embryo dataset through Entropy Sorting verses through highly variable gene (HVG) selection by Meistermann et al. 2021. **B.** Silhouette plots comparing the clustering performance of cells into the cell identities determined by Stirparo et al. 2018 through supervised analysis. The silhouette scores obtained when using the 3700 ES selected genes and a correlation distance metric are consistently higher than all other cases, indicating that the cells have been more confidently assigned to the cell type labels given by Stirparo et al. 2018. **C.** Heatmap of cell similarities when using the HVG selected genes and euclidean distance. **D.** Heatmap of cell similarities when using the ES selected genes and correlation distance. C. and D. are a visual representations of how cell type identities can become more distinct when using the ES selected genes. The full workflow for choosing the ES highly structured genes and discussion around using euclidean or correlation distance metrics can be found in our online methods.

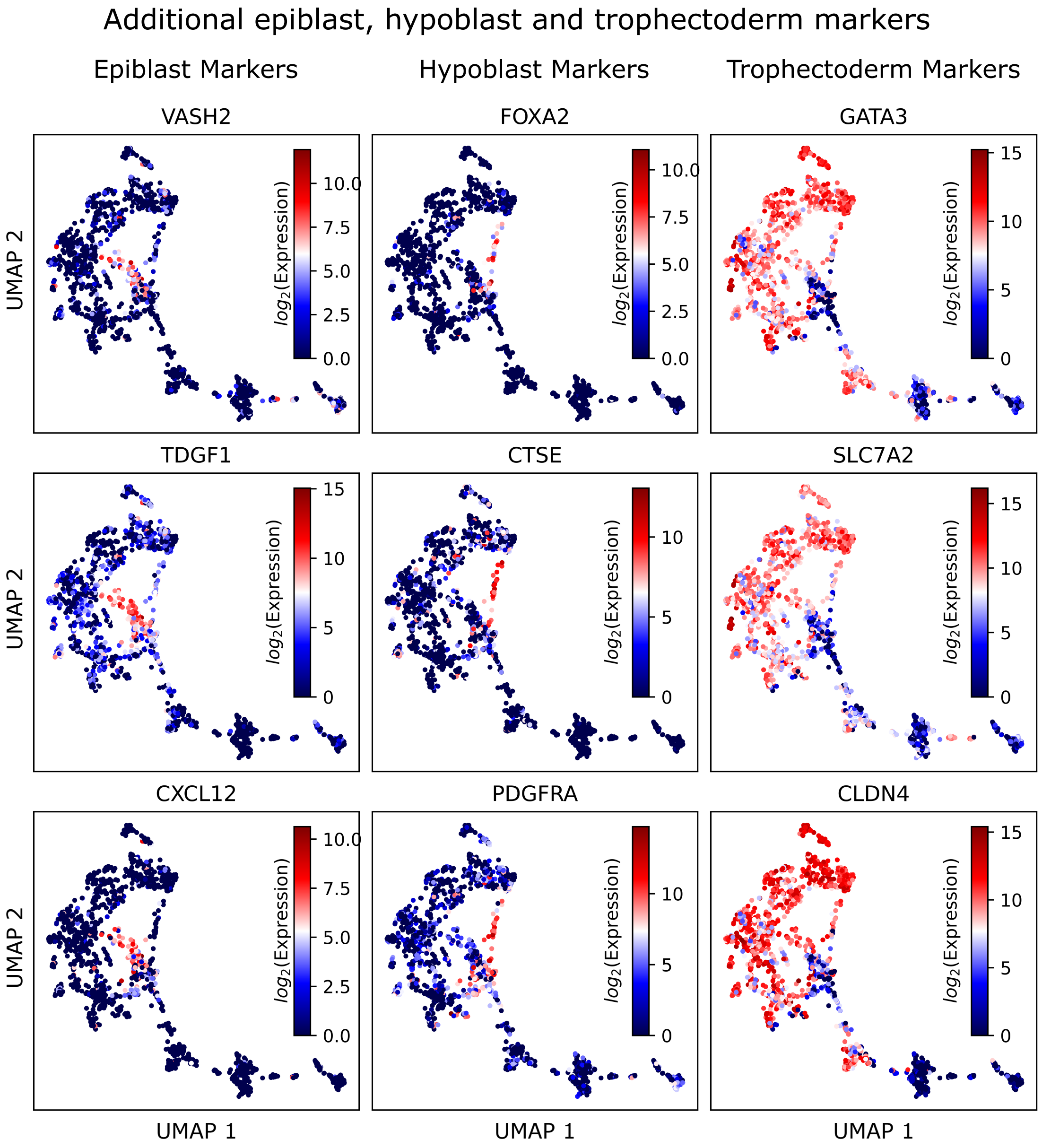

Figure S8. Additional epiblast, hypoblast and trophectoderm cell type marker expression profiles. Related to Fig. 6B.

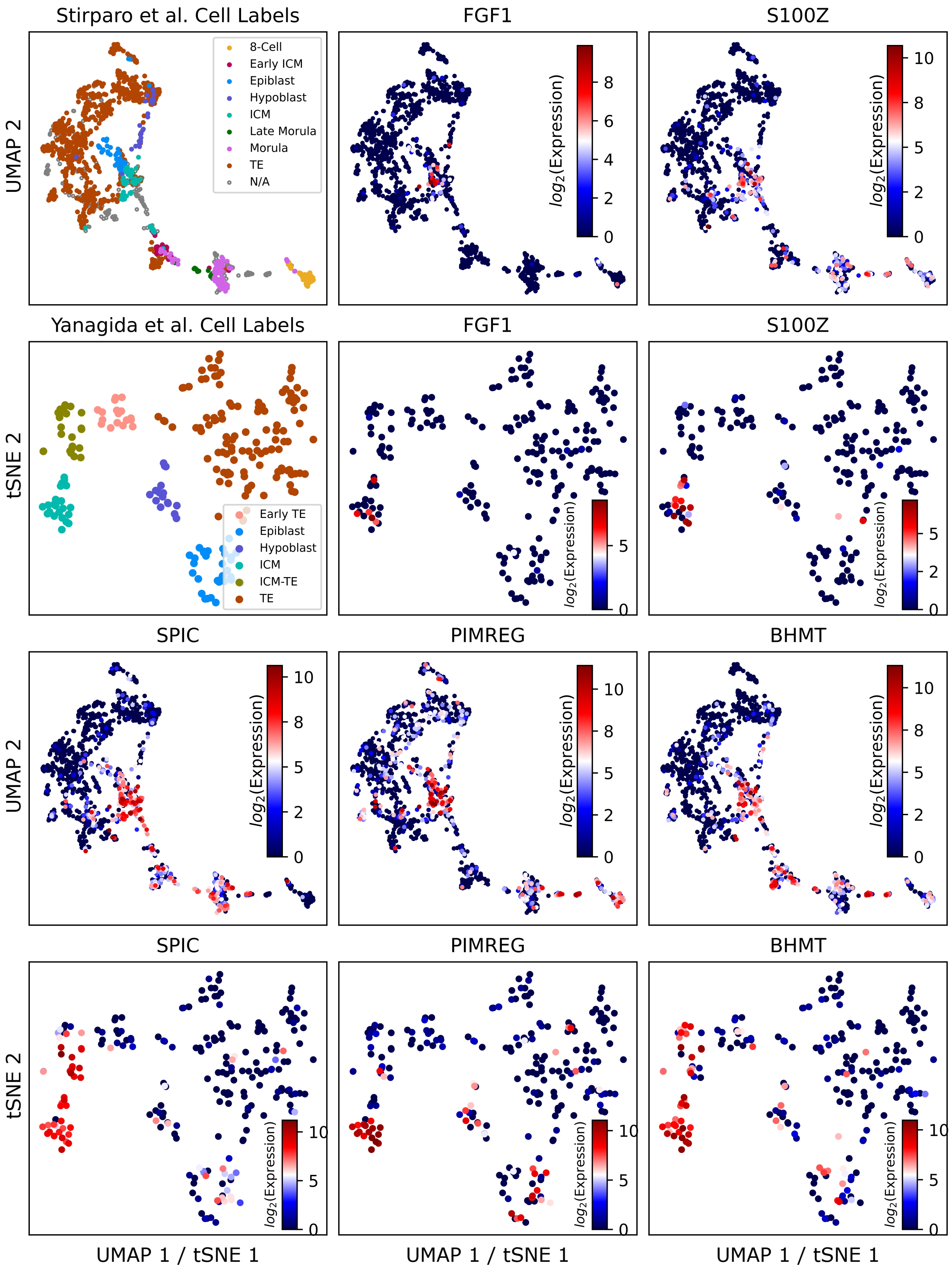

Figure S9. **Additional potential ICM marker expression profiles. Related to Fig. 7.** Potential ICM markers were selected based on their localised expression in the ICM population of our FFAVES + ESFW embedding that is corroborated in the UMAP embedding generated by Yanagida et al. on their own independent human pre-implantation embryo scRNA-sequencing data.

Antibody	Supplier	Catalogue number	Dilution
OCT4 (C-10)	Santa Cruz	#sc-5279<br>RRID:AB_628051	1:200
SOX17	R&D	AF1924<br>RRID:AB_355060	1:200
LAMA4	Invitrogen	# PA5-38938<br>RRID: AB_2555530	1:100
Donkey anti-goat Alexa Flour 488	Invitrogen	# A32814,<br>RRID: AB_2762838	1:500
Donkey anti-rabbit Alexa Flour 555	Invitrogen	# A-31572,<br>RRID: AB_162543	1:500
Donkey anti-mouse Alexa Fluor 647	Invitrogen	# A-31571,<br>RRID: AB_162542	1:500
Hoechst 33342	Invitrogen	H3570	1:1000

Table S1. Human embryo immunostaining antibody information.

Supplementary Information

SI 1 Discretisation of Continuous Data

A requirement for applying ES is that the data be discrete. We arbitrarily choose to represent these distinct states as 0's or 1's. How to discretise continuous gene expression data can be non-trivial. Consider the hypothetical gene expression profiles in Fig. SI 1. In the bimodal scenario (Fig. SI 1A), there are two distinct populations of gene expression. A large proportion of the cells display an expression value of 0, indicating that the gene is inactive. A second population display a normal distribution of expression with mean = 5 and standard deviation = 1. Hence, it could be considered reasonable that any data point with a value greater than zero corresponds to an active state and should be discretised to 1 (e.g. a discretisation threshold of 1, as indicated by the green dashed line, achieves this discretisation). However, from a biological standpoint, we may be sceptical as to whether low non-zero expression values represent a functionally active expression state. If they do not, we should discretise them to 0 to signify an inactive state (for example, using a discretisation threshold of 3.5, indicated by the dashed red line). The problem is further confounded if you consider multimodal data, for example if a gene is considered to have distinct functionality at more than two distinct expression states. In this case, as illustrated in Fig. SI 1B, we may wish to distinguish the different functionalities of the expression states defined by threshold 2 and threshold 3 (red and yellow dashed lines, respectively).

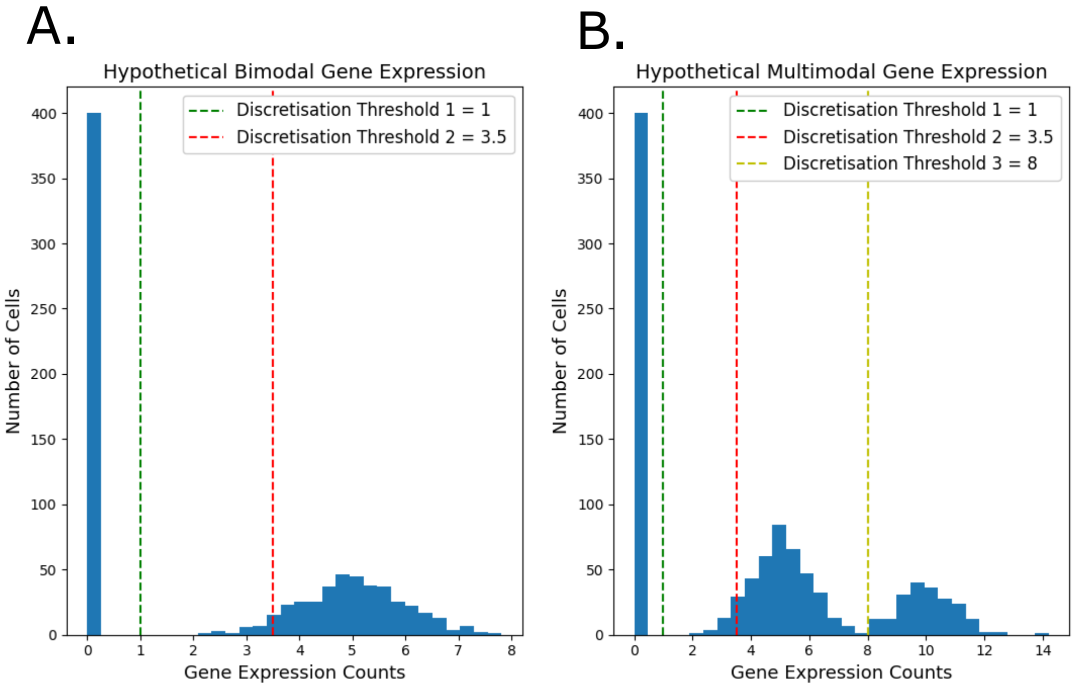

Figure SI 1. Choosing discretisation thresholds for continuous data. A. Example of a bimodal distribution for a given feature from continuous data with two possible suggested thresholds for discretisation. **B.** Example of a multimodal distribution for a given feature from continuous data with three possible suggested thresholds for discretisation.

Although it is a requirement that ES be presented with discrete data, this abstraction of the data can be justified in many scenarios. The strength of ES is the ability to identify discrete functional relationships between features in high dimensional data, rather than correlative patterns on the continuous scale. This is often a tractable and useful task. In the context of gene expression, we often talk about a gene being active or inactive, or being functionally related to other genes in the data, without specifically being interested in the raw expression values. Often we are simply interested in whether a gene's expression is distinctly different in one context versus another. To further emphasise that FFAVES and ESFW are tools to aid interpretability of data, having identified functional relationships between features in a data set using FFAVES/ESFW, we recommend that if the data was originally in a more complex format (i.e. a continuous scale), to subsequently return to this modality and use the information garnered through FFAVES/ESFW to enhance analysis.

How best to discretise the data is context dependent and left to the user. However we can simplify the procedure by demonstrating that as long as the discretisation is achieved reasonably well, and not necessarily perfectly, FFAVES has the capacity to automatically adjust and optimise the discretisation threshold of each feature based on the prevailing structure in the

data (Fig. 4g).

For now, consider the bimodal example presented in Fig SI 1A. Either of Threshold 1 or 2 constitute reasonable levels to discretise the data, depending on assumptions. Let us now assume that we chose Threshold 2, but the ground truth of the data actually agrees with Threshold 1. In this scenario, we would assign every data point with a value greater or equal to 3.5 the expression state 1 and every observation less than 3.5 an expression state of 0. Since the ground truth is that all values greater than 0 constitute a functionally active expression state, it follows that all values less than 3.5 but greater than 0 are FNs. Hence, when we perform ES on this feature, the FN data points will be quantified as divergent. If there is enough evidence for this divergence to be statistically significant, the expression states of the FN points will be switched from 0's to 1's. This is equivalent to lowering the discretisation threshold. If this process were to perfectly adjust the threshold, FFAVES would automatically adjust the threshold from Threshold 2 down to Threshold 1.

Finally, to address the issue of multimodality, consider the example of a feature displaying a multimodal expression distribution from Fig. SI 1B. There are two perspectives from which FFAVES can be found to deal with multimodal functionality. First, while there may be scenarios where FFAVES will struggle to directly elucidate the subsets of features that are related to separate functional modalities for a feature of interest, the resulting substructure can still be identified. For example, we could reasonably hypothesise that there exists a set of genes (S_1) that are functionally activated when the feature has an expression value between 1 and 8 (red line to yellow line). We could then also postulate that there is a second set of genes (S_2) that only become functionally active when the expression of the given gene is greater than 8 (above yellow line). If we were to discretise the given gene at a threshold of 1, then it is unlikely ES would identify the genes that are members of S_1 or S_2. This is because their functionally active states would span distinctly different regions of the data. However, this issue is mitigated by the fact that if the genes within S_1 are functionally related to each other in a distinct region of the data, ES will identify these genes as forming a distinct functional module. Likewise for S_2. Hence, although FFAVES would fail to identify that the genes within S_1 or S_2 are functionally related to distinct modalities of the multimodal feature in Fig. SI 1B, the information regarding the genes within S_1 and S_2 existing in a distinct region of the data is still captured. This process is exemplified in the synthetic data within the main body of this paper through the 50 multimodal genes (Fig. 4a).

An alternative method for discretisation exists, which may be desirable if a user is confident that features in the data exhibit functional multimodal activity, and they would like to identify these cases. In this scenario, the user may wish to create discrete 'pseudo-features' before inputting them into FFAVES. In Fig. SI 1B this could be achieved by turning the multimodal feature into 3 new discrete features. The first pseudo-feature (PF_1) could have all values greater than 1 discretised to 1 and all other values equal to 0. The second pseudo-feature (PF_2) could have all values between 1 and 8 discretised to 1 and all other values equal to 0. Finally, the third pseudo-feature (PF_3) could have all values greater than 8 equal to 1 and all other values equal to 0. Now ES would find features that become functionally active whenever the feature of interest is expressed with a value greater than 0 as being functionally related to PF_1. Likewise the genes within S_1 and S_2 would be identified as being functionally related to PF_2 and PF_3 respectively. Once again, if such a threshold technique was carried out reasonably well, FFAVES should be able to automatically optimise the thresholds used to create each pseudo-feature. However, it should be noted that seeking higher resolution in this manner for many features could dramatically increase the number of observed features in a given data set and thereby increase the computational run time.

SI 2 Deriving the Entropy Sort Equation

In the following we derive the Entropy Sort Equation (ESE). To do so we use the toy example in which the expression profile of 30 cells is measured, and consider the activity of two genes. We state that in $Gene_1$, 10 of the samples display the minority state and in $Gene_2$ 8 of the samples express the minority state. We seek to quantify to what degree the expression of one gene correlates with the expression of the other.

We treat this as a sorting problem, and first discretise the expression of each gene. In our example, if we inspect either Gene 1 or Gene 2, we can calculate their Shannon Entropy (Shannon 1948), H, as follows:

$$H = \sum_{i=1}^{n} -p_i \log_2(p_i), \tag{1}$$

where p_i is the probability of selecting a particular expression state i in the samples and n is the number of unique states (here, $n = 2$ since there are two discrete states). For Gene 1, 10 cells display Gene 1 as active and 20 display Gene 1 as inactive. Hence, the entropy for Gene 1 is given by

$$H_{Gene\ 1} = -\frac{10}{30}\log_2(\frac{10}{30}) - \frac{20}{30}\log_2(\frac{20}{30}) = 0.918. \tag{2}$$

Likewise, the entropy for Gene 2 is calculated to be

$$H_{Gene\ 2} = -\frac{8}{30}\log_2(\frac{8}{30}) + -\frac{22}{30}\log_2(\frac{22}{30}) = 0.837. \tag{3}$$

The Shannon Entropy of a given variable describes the average amount of “information" or “uncertainty" inherent in the variable’s possible outcomes. Up to this point Gene 1 and Gene 2 are considered as two independent variables. To extend Shannon Entropy to quantify the relationship between the two genes, we set up the following hypothesis. We assume that splitting the data into two groups based on the expression of Gene 1 is a perfect partition of disorder in the system. That is, if we group all cells for which Gene 1 is active and then separately group all the cells for which Gene 1 is inactive, the expression states of every other gene in the data would also be perfectly sorted into these two groups. We then test how true this is for the other genes in the data, i.e. Gene 2.

We designate group 1 (G_1) to contain those cells in which Gene 1 is active, and group 2 (G_2) to contain those cells in which it is inactive. This minimises the entropy of the system to zero, since both groups are homogeneous. We now maintain the Gene 1 groupings while inspecting the expression states of Gene 2. These groups fail to perfectly sort the expression states of Gene 2, since the number of minority states of gene 2 cannot be equal to the cardinality of group 1 or group 2 of the reference feature. To quantify this, we calculate the entropies of G_1 and G_2 with regards to Gene 2. For G_1, there are 10 cells in total, 5 of which display Gene 2 as active and 5 display Gene 2 as inactive. Therefore,

$$H_{G_1} = -\frac{5}{10}\log_2(\frac{5}{10}) + -\frac{5}{10}\log_2(\frac{5}{10}) = 1.00. \tag{4}$$

Likewise for G_2, since 3 cells show Gene 2 as active and 17 as inactive, the entropy is given by

$$H_{G_2} = -\frac{3}{20}\log_2(\frac{3}{20}) + -\frac{17}{20}\log_2(\frac{17}{20}) = 0.610. \tag{5}$$

It follows that the total entropy of the system is 1.610. To account for the different cardinalities of G_1 and G_2 we add a weight term for each calculation. In doing so we acknowledge that the entropy of G_1 and G_2 are portions of the entropy of the entire data set, and ensure that entropy of the system will always be a value between 0 and 1. Hence,

$$\text{Gene 2 System Entropy} = \frac{|G_1|}{C_T}H_{G1} + \frac{|G_2|}{C_T}H_{G2} = \frac{10}{30} * 1 + \frac{20}{30} * 0.610 = 0.740. \tag{6}$$

C_T designates the total number of cells in the system ($C_T = 30$) and $|G_1|$ and $|G_2|$ are the cardinalities of G_1 and G_2 respectively.

Intuitively, Eqn 6 corresponds to the conditional entropy of Gene 2 given Gene 1. In other words, given the expression states of Gene 1, it defines the uncertainty around the expression states of Gene 2 in any particular cell. Typically conditional entropy is considered as purely probabilistic, resulting in a single value between 0 and 1 that quantifies the dependency between two or more random variables. We seek a similar quantification of the relationship between Gene 1 and 2, but as a sorting problem. To achieve this, we note that there are optimal arrangements where the entropy of the system defined by the expression states of

two genes can be minimised. To minimise the conditional entropy of an observed system between two genes we must first constrain the system around one of the genes, Gene 1. The expression states of Gene 1 define two groups of fixed size. We then inspect Gene 2, and permute the locations of the expression states of Gene 2 into an optimal arrangement that minimises the entropy of the system. As with the expression states of Gene 1, the number of active/inactive states of Gene 2 is fixed to the value initially observed in the data, and it is simply their abundance within G_1 and G_2 that may be changed.

To highlight that entropy sorting generalises beyond scRNA-seq data, moving forward we will refer to genes as features. We designate the feature that defines groups 1 and 2 as the Reference Feature (RF), and any feature that is subsequently inspected as the Query Feature (QF). We will now formulate a smooth function that describes a range of conditional entropies for any RF/QF pair.

First, we outline some terms to generalise the ESE to any system. In the toy example that we have been using so far, we conveniently set up the system such that the number of cells with Gene 1 or Gene 2 active was smaller than the number of cells with the same gene inactive. To formulate the ESE as a sorting problem we can envision the sorting task as enriching the overlap of less commonly observed states, which in our example would be the active gene expression states. Alternatively, we could view the sorting problem as enriching the overlap of the more commonly observed states, but in either scenario the conditional entropy is the same. The practical difference is simply whether you choose to count how many of the less commonly observed states of the RF/QF pair overlap, or how many of the more commonly observed states overlap.

To this end, we employ notation to indicate whether the sample is displaying the less common or more common state for a given feature. We designate the more common state as the Majority (M) state, and the less common state the Minority (m) state. Hence, for any RF/QF pair, the minority state of the RF refers to those samples that form Group 1 (G_1) when partitioning the data and the majority state of the RF refers to samples that form Group 2 (G_2). Having partitioned the data via the RF, we can formulate terms describing how the QF is arranged within G_1 and G_2. Here, QF_{m,G_1} and QF_{M,G_1} correspond to the number of minority and majority QF states that occupy G_1, respectively. Similarly, QF_{m,G_2} and QF_{M,G_2} represent the number of minority and majority QF states in G_2.

We return to Eqn 4 and Eqn 5 to reform the calculations for the entropies of G_1 and G_2 such that

$$H_{G_1} = \frac{|G_1|}{|G_1|+|G_2|}\left(-\frac{QF_{m,G_1}}{|G_1|}\log_2\left(\frac{QF_{m,G_1}}{|G_1|}\right)-\frac{QF_{M,G_1}}{|G_1|}\log_2\left(\frac{QF_{M,G_1}}{|G_1|}\right)\right), \tag{7}$$

$$H_{G_2} = \frac{|G_2|}{|G_1|+|G_2|}\left(-\frac{QF_{m,G_2}}{|G_2|}\log_2\left(\frac{QF_{m,G_2}}{|G_2|}\right)-\frac{QF_{M,G_2}}{|G_2|}\log_2\left(\frac{QF_{M,G_2}}{|G_2|}\right)\right). \tag{8}$$

Equations 7 and 8 allow us to calculate the entropy of G_1 and G_2 based on the observed arrangement of any QF with respect to a given RF. The conditional entropy is then the sum of H_{G_1} and H_{G_2}. The highlighted terms emphasise the dependent variables. We can re-write three of these variables such that they are each functions of (QF_{m,G_1}):

$$QF_{M,G_1} = |G_1| - QF_{m,G_1}, \tag{9}$$

$$QF_{m,G_2} = QF_m - QF_{m,G_1}, \tag{10}$$

$$\begin{aligned} QF_{M,G_2} &= |G_2| - QF_{m,G_2} \\ &= |G_2| - (QF_m - QF_{m,G_1}) \\ &= |G_2| - QF_m + QF_{m,G_1}. \end{aligned} \tag{11}$$

Here we have included a constant term, QF_m, which describes the total number of QF samples that display the QF minority state (regardless of G_1 or G_2 overlap). Substituting into (7) and (8),

$$H_{G_1} = \frac{|G_1|}{|G_1|+|G_2|}(-\frac{QF_{m,G_1}}{|G_1|}\log_2(\frac{QF_{m,G_1}}{|G_1|})-\frac{|G_1|-QF_{m,G_1}}{|G_1|}\log_2(\frac{|G_1|-QF_{m,G_1}}{|G_1|})), \tag{12}$$

$$H_{G_2} = \frac{|G_2|}{|G_1|+|G_2|}(-\frac{QF_m-QF_{m,G_1}}{|G_2|}\log_2(\frac{QF_m-QF_{m,G_1}}{|G_2|})-\frac{|G_2|-QF_m+QF_{m,G_1}}{|G_2|}\log_2(\frac{|G_2|-QF_m+QF_{m,G_1}}{|G_2|})). \tag{13}$$

Finally, by summing (12) and (13), we derive the Entropy Sort Equation, which calculates the conditional entropy for any

RF/QF pair:

$$CE = H_{G_1} + H_{G_2}. \tag{14}$$

By defining the ESE to have only one variable, we have formed a continuous, bounded function. The curve produced by this equation represents a spectrum of conditional entropies that are defined by all possible arrangements of the QF with respect to the RF. In the main text we demonstrated that the resulting parabolic curve has a common structure for any RF/QF pair, with distinct properties that can give us new insights into the relationships between features.

One of the properties of the ESE that makes its use computationally tractable is that it is differentiable, such that;

$$\frac{dCE}{dx} = \frac{log_2(\frac{QF_m - x}{G_2}) - log_2(\frac{x}{G_1}) - log_2(\frac{G_2 - QF_m + x}{G_2}) + log_2(1 - \frac{x}{G_1})}{G_1 + G_2}. \tag{15}$$

Setting the left hand side of Eqn.(15) equal to zero and rearranging the equation we find,

$$x = \frac{|G_1| * QF_m}{|G_1| + |G_2|}. \tag{16}$$

By calculating the second derivative, we prove that this point is a maximum.

$$\frac{d^2CE}{dx^2} = -\frac{1}{G_1 + G_2}\left(\frac{1}{x} + \frac{1}{G_1 - x} + \frac{1}{QF_m - x} + \frac{1}{G_2 + QF_m + x}\right). \tag{17}$$

Since $x \geq 0$ (we cannot have less than 0 minority states overlapping), and $x \leq G_1$, $x \leq G_2$, $x \leq QF_m$, it follows that $\frac{d^2CE}{dx^2} < 0$, and the turning point is a maximum. Therefore, using Eqn. (16) we can calculate the maximum CE, from the value of x where the RF and QF are independent.

SI 3 Identifying the Reference Feature

In this section, we provide the rationale for determining which feature from a pair should be considered the RF or QF. In agreement with the established knowledge regarding conditional entropy, the ESE is non-symmetrical. In the notation of conditional entropy this corresponds formally to

$$H(RF_m|QF_m) \neq H(QF_m|RF_m). \tag{18}$$

We can view the asymmetry graphically if we take two example features and plot the ES parabolas generated when each feature is assigned as the RF (Fig. SI 2A, B). As expected, if the minority states of each feature have different cardinality, the ESE parabolas do not overlay with each other. However, they do have key common properties, such as existing within the same domain and having identical shapes. In fact, the two curves are simply linear transformations of one another along the y-axis. The curves exist within the same domain since the number of possible overlapping states between both features is encapsulated by the feature with the smaller minority state group. The magnitude of the transformation along the y-axis between the two curves can be identified by inspecting the maximum of each curve. Since the maximum is equivalent to each feature being independent from one another, the distance between the two curves is the difference in entropy:

$$\text{y-axis transformation} = |H(Feature\ 1) - H(Feature\ 2)|. \tag{19}$$

For any ES parabola, the QF is that which describes the possible rearrangements of the system and hence the conditional entropy along the ESE parabola. From this we can infer the important property that for any pair of features, the curve with the maximum entropy will be described when the feature with the larger minority state group is the QF.

Having identified the relationship between the two ES parabolas generated from a pair of features, we now consider the consequence of allowing either feature to act as the RF. Quantifying the correlation between two features will produce different values based on which feature is the RF, leading to potential asymmetry in the results. For example, if we were to take a set of features and create a matrix of feature correlations via Pearson's Correlation, the resulting matrix would be symmetric. This has practical consequences such as allowing the matrix to be used as a distance matrix for downstream analysis. Conversely, if we were to fix the RF for each row of the correlation matrix when calculating the ESS (Eqn. (4)), the upper and low triangles of the resulting matrix would not be equal. This would arise because the SW (Sort Weight) terms of the ESS would be different for a pair of features depending on which was the RF. This is because the weight term is dependent on the maximum of the ESE parabola, whereas SD and SG are not.

From the perspective of ES hypothesis testing, ambiguity around which feature is the RF further complicates the analysis. A critical example is when calculating EP for a pair of features. Since the ESE parabolas have identical shapes regardless of which feature is the RF, $DPC_{Dependent}$ (Eqn. 6) is equal for both ESE parabolas and so the assignment of the RF has no effect. However, $DPC_{Independent}$ (Eqn. 7) changes depending on which feature is assigned as the QF, since the ESE parabola maximum moves along the y-axis. Hence, if we do not identify a rationale to determine which feature is the RF in any pair, we cannot quantify errors across an entire data set without ambiguity.

To address the problem of RF assignment we apply the Principle of Maximum Entropy (Jaynes 1957a; Jaynes 1957b). The Principle of Maximum Entropy (MaxEnt) (Jaynes 1957a; Jaynes 1957b), states that given data and some constraints on a system, the probability distribution with the maximal entropy that satisfies those constraints should be chosen to represent the underlying data. We can relate MaxEnt to the problem of identifying the best distribution that describes an ES system. When setting up an ESE parabola for a pair of features, we have two distributions to choose from. Each distribution is constrained to the same domain and their maxima occur at the same value of x. MaxEnt states that we should always pick the distribution with the higher maximum entropy, which we have already identified as where the feature with the larger cardinality of minority states is designated the QF. Typically, identifying the distribution with maximum entropy would have to be approximated using mathematical approaches such as Lagrange Multipliers, as in more general systems there could be an infinite number of distributions that would satisfy the given constraints. However, we are fortunate that for our purposes there are only two possible distributions.

We can further justify the application of MaxEnt through Lesne's work on the Entropy Concentration Theorem (Lesne 2014). The Entropy Concentration Theorem rigorously quantifies the observation that when the number of samples in a data set is sufficiently large, the number of microscopic states (i.e. RF/QF pairs) underlying the MaxEnt distribution is exponentially larger than under any other distribution. The implication of this is that for a given data set with defined constraints, it is exponentially less likely that the observed values were sampled from any distribution other than the MaxEnt one.

We can demonstrate this with regards to ES via a simple example (Fig. SI 2A). Let the number of samples ($N = 20$) be small enough such that we can apply brute force to identify every microscopic state. We then observe two features, $F1$ and $F2$. $F1$ contains 5 minority states ($F1_m = 5$) and $F2$ contains 8 minority states ($F2_m = 8$). As expected, if we plot the two

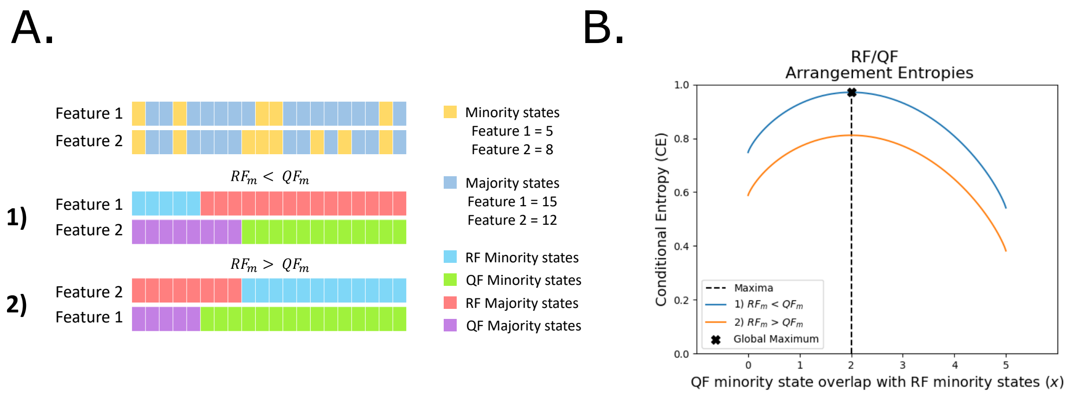

C. RF/QF Microscopic State Summary

	Microstate Cardinality	Minority State Overlap Frequencies						
		$x = 0$	$x = 1$	$x = 2$	$x = 3$	$x = 4$	$x = 5$	Sum
**1)** $RF_m < QF_m$	$\binom{20}{8} = 125970$	6435	32175	50050	30030	6825	455	125970
**2)** $RF_m > QF_m$	$\binom{20}{5} = 15504$	792	3960	6160	3696	840	56	15504
								Sum
Overlap Probabilities:		0.051	0.255	0.397	0.238	0.054	0.004	1.0

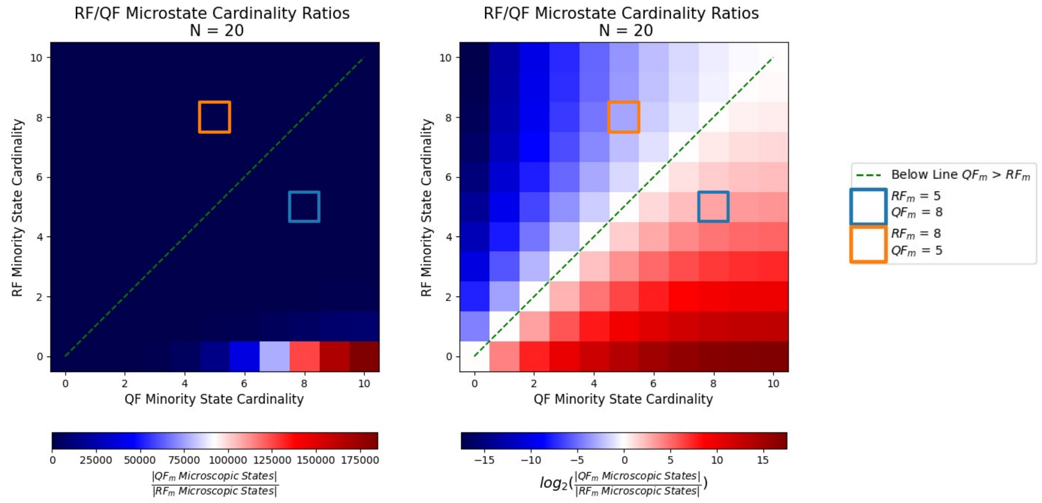

Figure SI 2. The maximum entropy principle informs which feature should be the RF. A. A simple example in which we could consider the feature with fewer minority states as the RF (1) or the feature with more minority states as the RF (2). **B.** The ESE parabolas formed from (1) or (2) from A. **C.** Summary of the frequency distributions for the observed RF/QF minority state overlaps (x) that could occur from all possible arrangements of the QF for (1) and (2). **D.** Heatmaps showing the ratio of all possible QF minority states vs. all possible RF minority states changes exponentially as the difference in cardinality of the RF/QF minority states increases linearly.

possible ES parabolas, the parabola with the global maxima occurs when $F2$ is the QF (Fig. SI 2B). ES requires us to fix the arrangement of samples based on the RF and then consider all permutations of the QF, observing the frequency of overlaps between the RF_m and QF_m states. The number of microscopic states (M) for any RF/QF pair is easily obtained as the number of possible arrangements of the QF minority states. Hence,

$$M = \binom{N}{QF_m}. \tag{20}$$

We can reformulate Eqn 20 in terms of M_x, the number of microscopic states corresponding to each possible observable overlap of minority states, x, within the domain of the ESE such that,

$$M = \sum_{x=0}^{RF_m} M_x = \sum_{x=0}^{RF_m} \binom{RF_m}{x} \binom{N - RF_m}{QF_m - x}. \tag{21}$$

We summarise the results of Eqn. 21 for our example in Fig. SI 2C. First observe that the total number of possible microscopic states when the $RF_m < QF_m$ is much larger than when the $RF_m > QF_m$ (125970 vs. 15504). This is in agreement with Lesne's Entropy Concentration Theorem (Lesne 2014), stating that the probability of observing a particular value of minority state overlap through sampling the MaxEnt distribution is exponentially more likely than from the other distribution. Hence, we should select the MaxEnt distribution as the best description of the system.

To further confirm that the two possible distributions are constrained in the same manner, if we count the frequencies of each value of x (Fig. SI 2C) amongst all possible microscopic states, we find them to be in equal proportions for both distributions. As such, the probability of each observation of x is equal, regardless of which distribution you choose, demonstrating equivalency. Finally in Fig. SI 2D, we illustrate how quickly the exponential nature of the Entropy Concentration Theorem is realised by presenting the ratio of RF/QF microscopic state cardinalities when we vary the value of RF_m and QF_m. Notice that even at a small value of N ($N = 20$), the number of possible microscopic states when $QF_m > RF_m$ is orders of magnitude higher than when $QF_m < RF_m$. This exponential increase is even more dramatically amplified as N increases, making it a strong rationale for selecting the MaxEnt ES parabola over the lower entropy parabola.

In summary, we demonstrate how the Principle of Maximum Entropy may be applied to the ES framework to preferentially choose the ESE parabola with the maximum entropy over the alternative ESE parabola. Being able to demonstrate that not using the MaxEnt distribution in our hypothesis testing would lead to testing results from different distributions removes ambiguity around which feature should be the RF and which should be the QF. The practical need to reject the lower entropy parabola becomes apparent in the context of ES hypothesis testing and algorithms, FFAVES and ESFW.

SI 4 FFAVES

Algorithm 1: FFAVES

```
Input   : M, m_min, CI, T, Max_Cycles
Output  : Suggested Divergent Points
begin
    i = 0
    while i ≤ Max_Cycles & ΔFN < T do
        M' = M    # Always start from initial discretised matrix
        if i > 0 then
            # Switch FN states learned from previous cycle
            M'[FN_{i-1}] = (M'[FN_{i-1}] × -1) + 1
        end
        Step 1: Identify False Positive (FP) data points
          1) Calculate FP error potential matrix
          2) Find statistically significant FP divergent indices
          3) Temporarily switch states of FP data points
             M'[FP] = (M'[FP] × -1) + 1
        Step 2: Identify False Negative (FN) data points
          1) Calculate FN error potential matrix
          2) Find statistically significant FN divergent indices
          3) Switch states of FN data points
             M'[FN] = (M'[FN] × -1) + 1
        Step 3: Identify spurious suggested False Negative (FN) data points
          1) Calculate FP error potential matrix
          2) Find statistically significant FP divergent indices
          3) Null FN data points that also appear as FPs
        Save suggested FP and FN indices
    end
end
```

In this section we present and describe the pseudocode for the FFAVES algorithm. FFAVES formulates theory from ES into a workflow that takes discrete high dimensional data and iteratively switches the expression states of those data points that appear to be in the wrong state until the system converges. There are multiple ways in which this task could be formulated and hence FFAVES is just one potential workflow for implementing ES. Additionally, recall that we derived the ESE such that x designates how many RF minority states overlap with QF minority states (SI 2). Hence, in this arrangement of the ESE a FP always designates when a data point displaying a minority state of a given feature should instead display the majority state. Conversely, a FN indicates that a sample displaying the majority state of a feature should instead display the minority state.

A summary of the nomenclature of the FFAVES pseudocode is as follows. M = input discrete state matrix, M' = discrete matrix augmented by suggested divergent points, m_{min} = minimum minority state cardinality for an accepted feature (default = 10), CI = confidence interval for identifying divergent points (default = 0.99), T = tolerance for convergence (default = 0.1%), Max_Cycles = maximum number of cycles before terminating FFAVES (default = 15), i = cycle number, FN = suggested false negative data points, FP = suggested false positive data points, ΔFN = change in number of suggest FN data points between cycle i and cycle $i-1$ as a percentage of the number of data points in M.

It is useful to further clarify a few symbols.

- m_{min}: The minimum cardinality for an accepted feature, i.e. the minimum number of minority states a feature can have before it is excluded from analysis.

  The default value of $m_{min} = 10$, such that features with fewer than 10 data points in the minority state will be ignored when running FFAVES. The default value is a relatively arbitrary cutoff, but we provide an empirical motivation for selecting a default of $m_{min} = 10$ in Section. SI 6. There we demonstrate that for $m_{min} < 20$, the sensitivity of a feature to FN data points reaches a point where ES hypothesis testing automatically detects that there is not enough information to suggest FNs through positive EP values (Eqn. 8). Hence, ES hypothesis testing incorporates quality control checks that will tend to be automatically enforced at a value of $m_{min} > 10$, further demonstrating the arbitrary nature of the m_{min} parameter.

- CI: The confidence interval. A threshold used to determine whether the EP values of individual data points are statistically

unlikely to be part of a half normal distribution of all calculated EP scores.

The default value of $CI = 0.99$, such that EP values found outside this confidence interval have only a 1% chance of being a member of the distribution. As such, we can confidently say that these data points are anomalous and suggest them to be FP or FN expression states.

- T: The tolerance. A threshold for when the expression states of the FFAVES adjusted matrix (M') have converged, and FFAVES may terminate before reaching the maximum number of iterations defined by Max_Cycles.

  The default value of $T = 0.1\%$, such that if the change in the number of suggested FN data points between cycle i and cycle $i-1$ is less than 0.1% of all data points in M' for three cycles in a row, FFAVES will terminate early.

The FFAVES pseudocode shows that FFAVES has three main steps.

1. Identify statistically significant FP data points and switch their states in the discretised matrix (M).

2. Identify FN data points in the adjusted state matrix (M'), followed by a second state switch of data points that appear in the wrong state due to being FNs.

3. Repeat step 1 on M' to identify further possible FP data points. If any of the suggested FPs identified in step 3 were suggested as FNs in step 2, they are considered spurious suggestions and removed from the set of suggested FN points.

The final set of FN data points are saved and applied to the data at the start of the next iteration. This continues until the algorithm reaches the maximum number of cycles or converges within the tolerance (T) limit.

The workflow outlined in the FFAVES pseudocode is an example of a specific application of ES. It is intentionally conservative in suggesting that the minority state of any feature be enriched. The motivation for this is that we are often primarily interested in how the presence of uncommon minority state observations distinguish samples from one another. In other words, real minority state expression states are information rich. Correctly identifying FN minority state expression states through ES amplifies the prevailing structure in the data. However, the unintentional introduction of FP minority states diminishes the value of the real minority states, potentially leading to a lower resolution of differing sample identities than if we had not tried to correct the expression states at all (Andrews and Hemberg 2019). For example, if shared gene expression states between distinct cell types through FPs, those cell types may end up looking more similar in the data than they really are.

To minimise the introduction of FP minority states, Step 1 aims to prune minority state expression states that appear significantly contradictory to the prevailing overlapping state structure in the data. This is important for Step 2, when FFAVES seeks to identify FN minority state data points. If the spurious minority state points from Step 1 are not pruned, they could provide evidence for suggesting FN data points with equal weighting as data points that have low potential of being FPs. Hence by temporarily removing likely FP data points in Step 1, we mitigate the possibility of poor quality data points generating more poor quality data points.

An example of this could be cells transitioning from one state to another. As cell identity evolves from cell type 1 (CT_1) to cell type 2 (CT_2), sets of genes are upregulated and downregulated. However, there may be a delay in the breakdown of some RNA species and we may detect expression of a gene specific to CT_1 in a sample belonging to CT_2. Failure to implement Step 1 in FFAVES could lead to ES suggesting that the sample from CT_2 should inherit a portion of the gene signature from CT_1. This is because FFAVES would quantify that it is rare to find the spurious CT_1 gene active in the absence of other CT_1 genes. By implementing Step 1 we minimise the likelihood of these pre-existing FPs events generating new FPs.

Step 3 is another quality control step designed to minimise the introduction of FPs. Once again consider that there was enough evidence in Step 2 to suggest that a gene specific to CT_1 should be expressed in CT_2, but it is being displayed as inactive (i.e. a FN). This may be due to stochastic overlapping gene signatures as the cell transitions between the two cell states. Now imagine that in Step 3 that same gene expression point is also found to be a FP due to the conflicting gene signatures. In other words, the data point is found to be both a FN and a FP. In this scenario, Step 3 identifies that there is conflicting information. Hence, we cannot make a judgement as to whether the data point should change state or not, so we remove the data point from the list of suggested FNs, thereby defaulting back to the expression state observed in the data.

Note that state switching of potential FPs in Step 1 is only a temporary removal of suggested FP states from the initial matrix (M). We choose only to switch the states of proposed FN points at the start of each cycle for two reasons: i) We believe that the presence of FP minority states in M is very low. This is primarily inspired by scRNA-seq data, where it is unlikely that FP data is introduced during the experimental generation of the gene expression matrix. We believe this is not unreasonable for many data sets, but it is up to the user to decide. Thus, if they are not FPs, but real biological data points due to factors such a gradual decay of mRNA fragments, we do not want to entirely remove them from the data as they may elucidate gene expression gradients/dynamics in the whole data set. Rather, we temporarily remove them to protect from the introduction of FPs by FFAVES. ii) By returning to M at the start of each cycle and only changing the states of suggested FN data points from

the previous cycle, FFAVES checks whether FPs identified in the previous cycle are no longer significantly out of place in the adjusted data. If so, they can confidently be used to amplify the prevailing structure in the data to further elucidate FN data points. One way to interpret this is that we are anchoring the convergence of the expression states around the information rich, high confidence minority states.

Calculating the error potential matrix

The first step for identifying FP or FN states is to calculate the Error Potential Matrix (EPM). The EPM can exist in two forms: the false positive EPM (EPM_{FP}) and the false negative EPM (EPM_{FN}). These quantify the likelihood of data points in M' being FP or FN respectively. Hence, EPM_{FP} is used in Steps 1 and 3 of the FFAVES algorithm, and EPM_{FN} is used in Step 2.

We visualise the process of generating an EPM matrix in Fig. SI 3A. The majority of ES theory is utilised in steps (1) and (2). Step (1) draws upon the logical deduction of 8 specific error scenarios where divergence could be observed under the assumption of dependence between two features (Fig. S1), and the application of the maximum entropy principle (Section SI 3), to determine which set of calculations should be undertaken to form the required EPM. Step (2) performs these calculations using ES hypothesis testing to quantify the evidence suggesting that data points in M/M' are displaying the wrong state. The process of identifying the evidence that states are FPs or FNs is carried out for each feature. Hence, each column, j, of an EPM corresponds to the evidence suggesting that each sample, i, of feature j in M' is presenting as the wrong state.

We now describe each step of generating the EPM in more detail. Steps (1)-(4) in Fig. SI 3A highlight the process for a single feature, j, which we designate to be the Fixed Featured (FF). The FF refers to when we inspect a specific feature in the data and seek to quantify the evidence that the samples in the FF are FPs or FNs. During the process of quantifying the likelihood of FPs or FNs, the FF may act as either the RF or QF during ES hypothesis testing (determined in step (1)).

Step (1) determines how ES hypothesis testing should be carried out between the FF and any other feature (Fig. SI 3B). Having designated the FF and SF, we first identify whether the FF should be the RF or QF by comparing the cardinality of the FF and SF minority states (FF_m and SF_m, respectively - see Section SI 3). Subsequently, we identify which of the 8 possible error scenarios (Fig. S1) any divergence observed in the FF/SF pair relates to. We only consider scenarios in which $QF_m > RF_m$ (Section SI 3), and thus only ever observe error scenarios 2, 4, 6 and 8. Having applied the maximum entropy principle, we use the split direction (SD, Eqn. (1)) to determine the appropriate error scenario. If the FF/SF pair corresponds to scenarios 2, 4 or 8, any observed positive EP can be attributed to FPs in the FF. Conversely, if the FF/SF pair corresponds to scenario 6, any observed positive EP can be attributed to FNs in the FF (Fig. S1). Having applied the logic in Fig. SI 3B to the FF and all other features in the data set, we retrieve j_{FP} and j_{FN}, which identify all features where the calculated EP indicates the likelihood of FPs or FNs in the FF, respectively. We then feed j_{FP} or j_{FN} into step (2) of the EPM calculation (Fig. SI 3A).

Step (2) performs ES hypothesis testing between the FF and every relevant SF, as designated by j_{FP} or j_{FN}. This produces a matrix with i rows for each sample and j_{FP} or j_{FN} columns for each SF (Fig. SI 3A). Accordingly, each element of this matrix represents the EP for ith sample in the FF when it is considered against the j_{FP}th or j_{FN}th SF.

Step 3) ignores all negative EP values by setting them to 0. ES tells us that negative EP values indicate that divergent cells in the FF/SF pair are more likely to have occurred due to feature independence than due to the introduction of error. Conversely, positive EP values indicate that divergent samples are more likely due to the introduction of error. We are interested in quantifying all the evidence available that suggests that observed states in the FF are erroneous.

Finally, in step (4) we sum the rows of the ES hypothesis test matrix, such that we are left with a vector of i samples. Each element in this vector represents all evidence in the data set that suggests sample i in the FF is currently displaying the incorrect state in M'. This vector then fills column j of the EPM.

Depending on whether the EPM_{FP} or EPM_{FN} matrix has been calculated, the next step of FFAVES is to identify if any of the states in M' are statistically likely to be observed in the wrong state by identifying if their corresponding values in the EPM are unusually high. Such data points are considered to be consistently out of place based on the evidence provided by structural relationships between any FF of interest and all the other features in the data.

Identify statistically significant divergent data points

Having generated an EPM_{FP}/EPM_{FN}, we now wish to identify states that are likely FPs/FNs. To explain our approach, we describe an example, which relies on the synthetic data presented in this paper (SI 7), containing 5 synthetic cell types. These cell types are distinguishable from one another by modules of highly structured synthetic genes.

In this example, we are specifically evaluating the EPM_{FN} from Step (2) of FFAVES. Before attempting to find relationships or structure within the EPM, it is important to understand the two **if** statements at the start of the pseudocode. When calculating EPM_{FP}, we are only quantifying the EPs of all data points in M' that display feature minority states. Conversely, when calculating EPM_{FN} we are only quantifying the EPs of data points that are displaying majority states. Thus, when we seek to compare the values observed in an EPM_{FP} or EPM_{FN}, we should only inspect the indices that are relevant to the possibility of FP or FN error, respectively. More precisely, when inspecting an EPM_{FP} (EPM_{FN}), we should only take the indices in M' that relate to feature minority (majority) states, since these are the only points where we can quantify the potential for FPs.

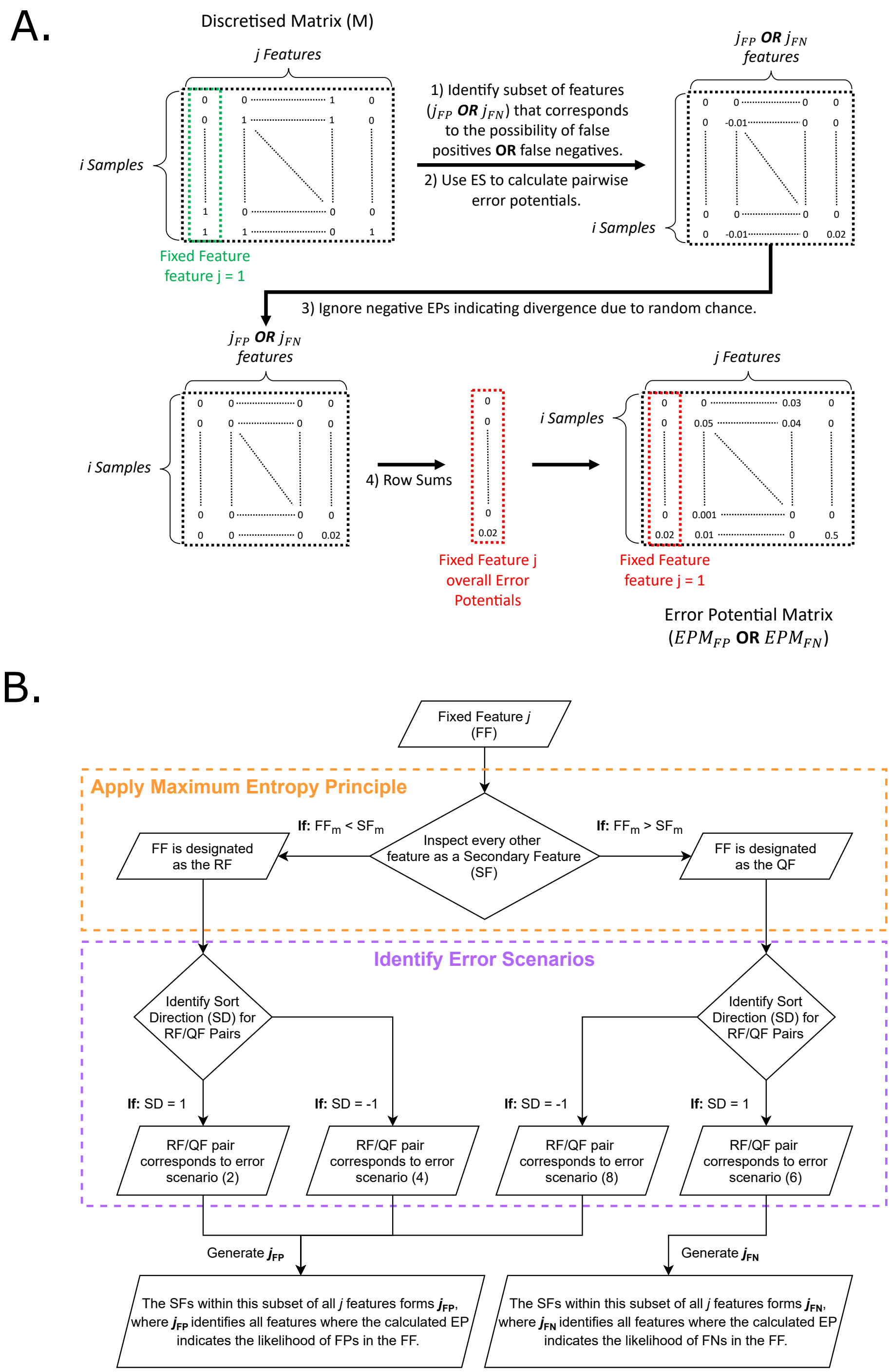

Figure SI 3. **A. Calculating an EPM.** The EPM is calculated by performing ES hypothesis testing in a pairwise fashion for every feature in the data. The EPM quantifies all the evidence available indicating that individual data points are in the wrong state in the discrete data matrix (M'). We summarise the calculation of an EPM in four main steps. **(1)** For each feature, identify all other features that would provide evidence that data points for the given feature are FPs/FNs. The identification of those features contained in j_{FP} or j_{FN} is summarised in Fig. SI 3B. **(2)** Perform ES hypothesis testing to calculate all EPs (Eqn. 8) between feature j and features j_{FP} or j_{FN}. **(3)** Set all negative values to 0 (we are only interested in scenarios where there is evidence that data points are in the wrong state due to the introduction of error). **(4)** Calculate the row sums of the positive ES hypothesis test matrix to get the total error potential evidence that the samples of feature j are displaying the wrong states in M'. **B. Identifying j_{FP} or j_{FN} for any given feature j.** For any feature in a data set, there exists a subset of features that have the potential to inform whether expression states in j are displaying as FPs (j_{FP}), and a subset of features that would suggest FNs (j_{FN}). Whether a secondary feature (SF) provides evidence for FPs or FNs is determined via whether the fixed feature (FF) has a larger or smaller minority state cardinality, followed by whether the FF and SF have split directions (SDs) equal to 1 or -1. Following this, for any FF we can identify all the features that are members of j_{FP} and/or j_{FN}.

Having identified which data points within a given EPM are to be analysed, we can now examine the distribution of EPM values to identify those that are statistically higher. Plotting a histogram of the EPM values reveals that the vast majority of potential FNs have EPM values around 0. There is also an exponentially smaller frequency of values within a given bin of the histogram as the EPM values increase. This provides a firm basis to model the distribution of the data as a half-normal distribution. This should be generally true for the majority of high dimensional data sets. If we observe a highly structured data set with little to no FN/FP data points, then all of the highly structured data points should have little to no error and hence have EPM values close to or equal to 0. Conversely, if we have a data set with little to no correlative structure between feature states in M, then the only scenarios we should see positive EP values during the calculation of the EPM would be by random chance. This in turn has a low chance of generating a high EP score, and hence the vast majority of scores in the EPM will be close to or equal to 0. By fitting the data to a half-normal distribution, we are then able to use the resulting cumulative distribution function (CDF) to identify when values are significantly larger than the distribution mean. Before we can fit the data to a distribution it is useful to convert the data into z-scores. The z-score of any data point within a distribution is a measurement of how many standard deviations above or below the population mean the original data point is. Transforming into z-scores is useful as it allows us to easily identify which values lie outside of a given confidence interval when the data is fitted to a half-normal distribution. We calculate the z-scores as follows:

$$z - score = \frac{x - \mu}{\sigma}, \tag{22}$$

where μ and σ are the mean and standard deviations of the data, respectively, and x is the value taken from the EPM. Since we are treating the data as a half-normal distribution, $\mu = 0$.

We fit the data to a half-normal distribution using the *halfnorm* function from the *scipy.stats* python package. This takes the z-scores as input, and returns where each z-score lies on the CDF. This converts each z-score into a probability of whether sampling from the distribution would produce a value less than or equal to the given z-score. From this we can form confidence intervals to identify when individual data points are statistically unlikely to be members of the observed distribution. We use a confidence interval (CI) on the half normal distribution of 0.99 which indicates that there is a 1% chance that if you were to sample from the fitted distribution you would observe a value greater than the respective z-score. Since it is statistically unlikely that observed z-scores outside this stringent CI are part of the observed distribution, we denote any points with higher z-scores as statistically divergent data points. In this example we consider an EPM_{FN} matrix, thus indicating that the statistically divergent points are FNs. The approach follows for EPM_{FP} matrices and FPs. Returning to the context of ES, identifying FPs/FNs data points as having statistically high EPM values is equivalent to stating that there is a significant body of evidence within the pairwise relationships of the features to suggest that the currently observed state in M' is incorrect. Having identified that which individual data points are likely FN or FP data points, we may confidently switch their expression state move to the next step of the FFAVES algorithm.

SI 5 ESFW

Algorithm 2: ESFW

```
Input   : M, FFAVES_Divergent_Indices, FN_Fraction, Iterations
Output : Feature_Weights
begin
    for i = 1 to Iterations do
        M' = M    # Always start from initial discretised matrix
        M' = (M'[FFAVES_Divergent_Indices] × 1) + 1    # Switch states
        for Each feature in M' do
            Randomly switch FN_Fraction of minority states to majority states
        end
        Calculate EPM_FN
        for Each feature in M' do
            Iteration_Weights = Mean of EPM_FN values greater than 0
        end
        Store vector of Iteration_Weights
    end
    for Each feature in M' do
        Feature_Weights = Mean of Iteration_Weights
    end
end
```

Here we present our algorithm Entropy Sorting Feature Weighting (ESFW). Using the divergent data points suggested by FFAVES and the rationale from ES to calculate EPM_{FN} matrices, ESFW assigns a weight to each feature in the dataset. Higher weights correspond to those pairs of features that have stronger correlation. Conversely, low weights indicate features with weak co-regulatory relationships, indicating that the distribution of their minority states is essentially random compared to other features in the data.

The first step of ESFW is to take the discrete state matrix (M) used as the input for FFAVES and create an augmented state matrix (M'), by switching the states of the indices that FFAVES suggested as significantly divergent. The next step is to intentionally add FN data points in the minority states of each feature in M'. Having started with the converged state matrix M', we can be confident that the majority of divergence observed by intentionally adding FNs is caused by the controlled introduction of the FNs rather than unknown factors. Hence, when we calculate EPM_{FN} and test whether relationships between dependent features gain significant divergence due to FNs, we can directly relate the observed positive EPs to features with strong dependent relationships. To ensure that the observed positive EPs are balanced, the same fraction of minority states, $FN\_Fraction$, are switched to the majority state for each feature. We set the default $FN\_Fraction = 0.1$, such that 10% of the minority state samples for each feature are randomly switched to the majority state. This default value was found empirically to produce good results over multiple data sets, and relatively stable when varied from 0.05-0.3 (data not shown).

To generate feature weights, we take the column means (columns correspond to features) for all points in EPM_{FN} that have values greater than 0. We inspect only EPM_{FN} values greater than 0 because these represent cases where introducing FNs has weakened feature dependencies through divergence. We take the column means of these EPM_{FN} values rather than the columns sums, to allow for the fact that features may be members of different sized correlated networks. For example, in scRNA-seq data, mRNA expression relating to GRNs that control one aspect of a cell's function/identity, e.g. its lineage, may be significantly smaller/larger than the GRN that identifies its lineage subtype. The introduction of FNs ($FN\_Fraction$) to random features will lead to zero or low average EPM_{FN} values.

Each of the column means obtained from EPM_{FN} is the weight for each feature in M. We repeat this process multiple times and the $Iteration\_Weights$ for each feature are saved. Multiple iterations are carried out to ensure the search space for introducing random FNs is well covered. The number of iterations undertaken is defined by the *Iterations* variable, with a default of 5. The final variable, $Feature\_Weights$, is a vector of length j and is the mean weight of each feature from $Iteration\_Weights$. Having obtained a vector of weights representing the importance of each feature in a given data set, we are then able to subset the data down to highly informative features, as demonstrated in the main text.

SI 6 ES Minority State Cardinality Sensitivity

In Fig. 4C and D we quantify and visualise the performance of FFAVES in identifying FNs. A closer look at the ground truth FNs we fail to be identify suggests that the majority occur in genes that are active in very few cells. This demonstrates one of the limitations of FFAVES. We hypothesise that as the number of samples displaying the minority state of a feature decreases, the sensitivity of that feature to FN drop outs increases. We can demonstrate this empirically with an idealised simulation.

For our idealised simulation, start with two perfectly overlapping features. We then randomly introduce a fixed fraction of FNs to each feature. Using ES hypothesis testing we calculate the error potentials (EP) of the divergent points between the features. After repeating this multiple times for different minority state feature cardinalities and for different fractions of FNs, we matrix of the average observed EP for each ideal pair of features. When we analyse this matrix of average observed EPs we find that as the minority state cardinality of a feature decreases, the average observed EP switches from positive to negative. Recall that a negative EP value indicates that there is no longer any evidence that the observed divergence was due to the introduction of erroneous observations. Hence, this agrees with our hypothesis that for a fixed rate of introduced FN error, the smaller the minority state cardinality of the feature, the more susceptible it is to having its co-regulatory dependencies disrupted beyond a point that ES can quantitatively identify the error introduced. After many simulations we identified an empirical guidance threshold that whenever a feature has less than 20 samples displaying the minority state, ES will become considerably less reliable in being able to correctly identify FN or FP data points.

SI 7 Synthetic Dataset Details

The synthetic data set that we derive is designed to reflect the major properties of scRNA-seq data while allowing us to have a well defined ground truth to interrogate. It was created by combining nine different properties that are typically observed in scRNA-seq data sets to create a synthetic data set that reasonably represents the challenges of real data. As such, it has no underlying model or simulation of gene regulation. The benefit of using a relatively simple methodology is that we can be precise about the ground truth of the data before we intentionally add error to test ES. This subsequently allows us to confidently quantify how well any particular method performs when trying to recapitulate the ground truth properties of the data. The nine properties of the synthetic data are described below ((ii)-(viii) are highlighted in Fig. SI 4A).

(i) **Continuous gene expression**: Whenever it is deemed that a cell should express a given gene, its expression value is sampled from a $N(5,1)$ distribution.

(ii) **Five synthetic cell types**: We initiate SD1 by creating five distinct 'cell types', which are each sets of 200 cells. Within each cell type there is a hierarchical structure of gene expression, such that the number of cells that a set of genes is expressed in decreases from 200 to 20 in increments of 10. That is, one set of genes is expressed in all 200 cells of the given cell type, followed by a set of genes expressed in 190 of the cells, and so on, until a final set of genes is only expressed in 20 of the cells. The size of each subset of genes is randomly sampled from a discrete uniform distribution with values between 5 and 15 genes. In this way, each cell type is identified by a set of tightly regulated genes. Simultaneously, within each cell type there contains easily interpretable heterogeneity through the sequential gradient of cell type specific gene expression.

(iii) **Multi modal gene activity**: A simple example of a set of 50 genes that are defined to be active in 2 of the cell types. However, in one of cell types the average expression is twice as high than in the other.

(iv) **Structured genes**: Of the 1519 genes in the synthetic data, 969 are considered to be the 'structured' genes, which are tightly regulated within the 5 cell types. The process of generating these tightly regulated modules of genes is outlined in item (ii).

(v) **Randomly expressed genes**: One of the inherent challenges of scRNA-seq is filtering out those genes that are poorly structured with regards to cell type or cell function (Ramskö Ld et al. 2009). In our synthetic data, we introduce a subset of 500 genes with random expression throughout all 5 cell types. For each gene we randomly select the proportion of cells that will express the gene from a $N(0.3,0.3)$ distribution, but only accept values between 0 and 1. That proportion of cells are randomly selected and their expression value is sampled from a $N(5,1)$.

(vi) **Leaky gene expression**: Each of the 5 cell types represents an idealised situation where genes are tightly regulated to define a cell identity. We introduce leaky gene expression to represent when genes are detected as having non-zero expression, but such gene activity is out of context according to the structure in the rest of the data. In reality this could occur due to poor gene expression control in the cell, or the cell transitioning from one state to another. To represent this in our synthetic data, we identified the number of cells where active gene expression was detected for each gene. We then randomly pick a value between 0.02 and 0.1 to choose what percentage of leaky gene expression to add to the data for that feature (2-10%). The number of leaky expression data points to be added to a particular gene is equal to the number of cells found to have the gene active, multiplied by the sampled value between 0.02 and 0.1. Leaky gene expression points are added by randomly switching that proportion of samples in the data from zero to non-zero expression values for each gene.

(vii) **Multiple cells per sample**: To incorporate the reality of a small number of samples with multiple cells (JD 2018) we added 200 cells (20% of the initial 1000 cells) made by randomly combining cells from the 5 synthetic cell types. For each multiplexed sample, the number of cells to be combined was determined by sampling from a Poisson distribution with mean = 1, but continuing to sample until a value greater than 1 was obtained. A Poisson distribution with mean = 1 is used in accordance with the methodology used when diluting a pool of cells to be sequenced (JD 2018). Having determine the number of cells in the multiplexed sample, that number of cells are randomly taken from the 5 synthetic cells types and summed together.

(viii) **Random up/down-regulation between cell types**: ES was developed with the aim of identifying functional rather than simply correlative relationships between features. To demonstrate this, we added a set of 50 genes that are randomly up-regulated in just two of the synthetic cell types. Although there is a higher frequency of observing these 50 genes in these two cell types, the likelihood of observing the active gene states overlapping with one another for the 50 features is still relatively low. To create the 50 genes, for each we randomly picked a fraction of the samples (2-10%) in the two

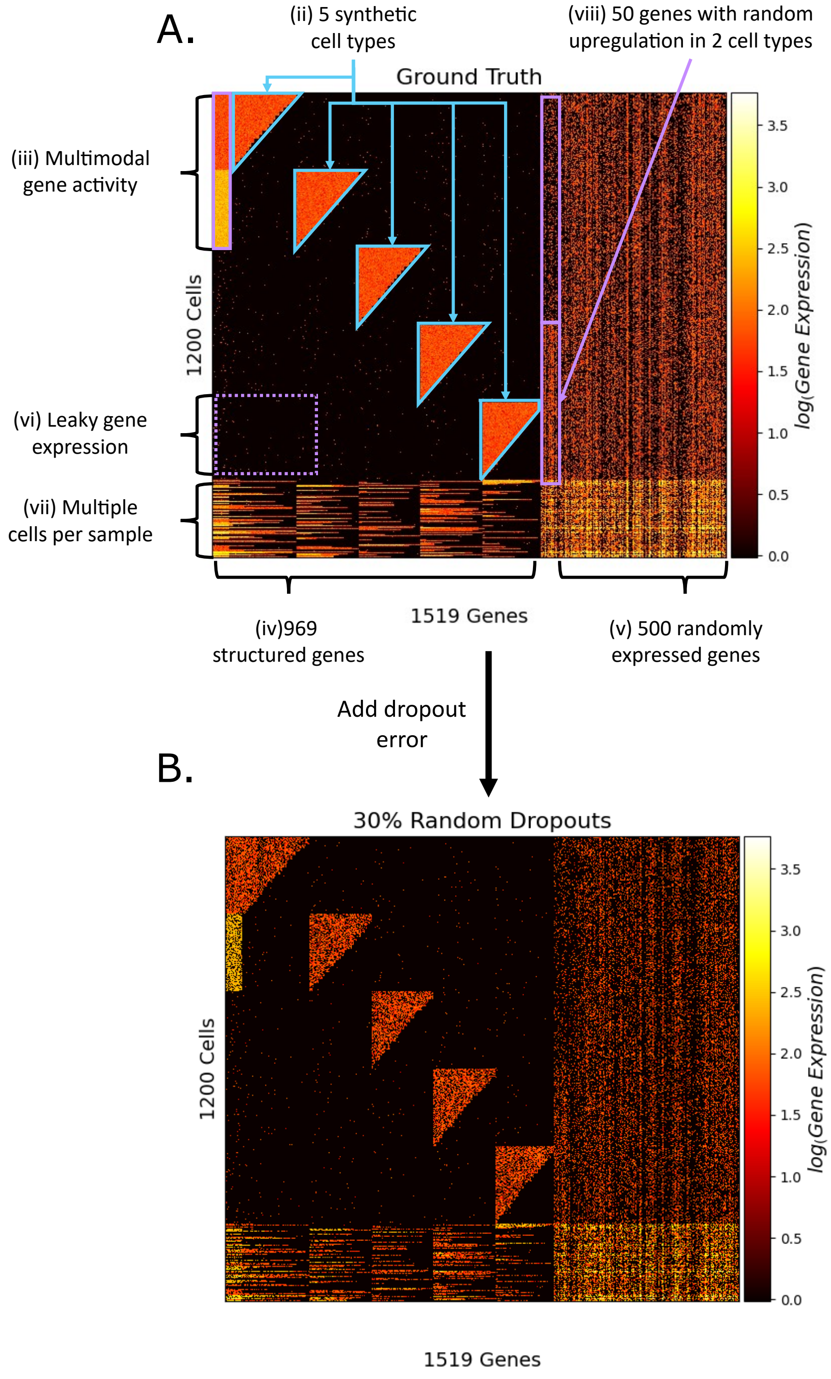

Figure SI 4. Synthetic data properties A. Visualisation of the ground truth synthetic gene expression matrix. Each of the defined properties of the data marked as (ii)-(viii) are described in Section SI 7. **B.** Visualisation of SD1 after 30% of the ground truth data points are converted to FNs (to mimic technical drop outs).

chosen cell types, and switched that fraction of cells to show the gene as active. For the remaining three cell types, we halved the fraction of cells showing the gene as expressed in the “up-regulated" cells and switched this smaller faction of cells to show the gene as active in the “down-regulated" cells. For example, if a gene would be active in 8% of the cells in the two cell types where it was up-regulated, the same gene would only display as active in 4% of the cells in the remaining three synthetic cell types.

(ix) **Batch effects**: Batch effects can be a significant confounding factor in the analysis and interpretation of scRNA-seq data. Having generated our synthetic dataset by combining each of the data properties in (i)-(viii), we add batch effects as follows. We create two batches of cells by grouping together odd and even numbered cells. For each gene we randomly select one of the batches and add a random drop out bias to all of the cells in that batch. This may be thought of as mimicking technical batch effects, where there is random variability in the capture efficiency of genes between the two batches. To add the drop out bias, we identify all cells in the batch in which the given gene is active. We take the absolute value after sampling from a $N(0,0.4)$ distribution to determine the fraction of active cells contained in the batch that will have their expression value set to 0 for the given gene. Finally, we randomly introduce the dropouts into the gene by switching the sampled fraction of cells from non-zero to zero values.

The culmination of each of these 9 synthetic data properties is shown in Fig SI 4A. This represents our known ground truth. We then add a final layer of complexity to the data by introducing a significant portion of FN random dropouts (Fig SI 4B). This is achieved by randomly selecting 30% of all data points and switching them to a value of 0 if they are not already 0. This noisy representation of the synthetic data serves as the starting point for us to try to recover the different substructures within the data, such as FNs/FPs and modules of highly co-regulated gene.

Supplemental Experimental Procedures

Human embryo immunostaining

Supernumerary frozen blastocysts (E5 and E6) were thawed and cultured in N2B27 medium under mineral oil until reaching the desired stage of development from E5 to E7. Embryonic stage was assessed based on thinning of the zona pellucida and blastocoele expansion.

The zona pellucida of D5 and D6 blastocysts was removed using acid Tyrode's solution before fixation with 4% PFA in PBS for 15 minutes at room temperature. Embryos were rinsed in PBS containing 3mg/ml polyvinylpyrrolidone (PBS/PVP), permeabilised using 0.25% Triton X-100 in PBS/PVP for 30 minutes and blocked in blocking buffer comprising PBS supplemented with 0.1% BSA, 0.01% Tween20 and 2% donkey serum for 2 hours at room temperature. Primary and secondary antibodies were diluted in blocking buffer (Table S1). Embryos were incubated in primary antibody solution overnight at 4 degrees and rinsed three times for >15 minutes each in blocking buffer before incubation in secondary antibody solution for 1-2 hours at room temperature in the dark. Embryos were rinsed in blocking buffer and imaged through a Poly-D-Lysin coated Mattek dish (P356-0-14) whilst submerged in blocking buffer. Embryos were imaged in a Leica Stellaris Confocal microscope and image analysis was performed using FIJI.

References

Andrews, Tallulah S. and Martin Hemberg (2019). “False signals induced by single-cell imputation”. In: *F1000Research 2019 7:1740* 7, p. 1740. DOI: 10.12688/f1000research.16613.2.

Cannoodt, Robrecht et al. (2021). “Spearheading future omics analyses using dyngen, a multi-modal simulator of single cells”. In: *Nature Communications 2021 12:1* 12.1, pp. 1–9. DOI: 10.1038/s41467-021-24152-2.

Davis, Jesse and Mark Goadrich (n.d.). “The Relationship Between Precision-Recall and ROC Curves”. In: *Proceedings of the 23rd international conference on Machine learning - ICML ’06* (). DOI: 10.1145/1143844.

Jaynes, E. T. (1957a). “Information theory and statistical mechanics”. In: *Physical Review* 106.4, pp. 620–630. DOI: 10.1103/PhysRev.106.620.

— (1957b). “Information theory and statistical mechanics. II”. In: *Physical Review* 108.2, pp. 171–190. DOI: 10.1103/PhysRev.108.171.

JD, Bloom (2018). “Estimating the frequency of multiplets in single-cell RNA sequencing from cell-mixing experiments”. In: *PeerJ* 6.9. DOI: 10.7717/PEERJ.5578.

Lesne, Annick (2014). “Shannon entropy: A rigorous notion at the crossroads between probability, information theory, dynamical systems and statistical physics”. In: *Mathematical Structures in Computer Science* 24.3. DOI: 10.1017/S0960129512000783.

Meistermann, Dimitri et al. (2021). “Integrated pseudotime analysis of human pre-implantation embryo single-cell transcriptomes reveals the dynamics of lineage specification”. In: *Cell Stem Cell* 28.9, pp. 1625–1640. DOI: 10.1016/j.stem.2021.04.027.

Ramskö Ld, D et al. (2009). “An Abundance of Ubiquitously Expressed Genes Revealed by Tissue Transcriptome Sequence Data”. In: *PLoS Comput Biol* 5.12, p. 1000598. DOI: 10.1371/journal.pcbi.1000598.

Shannon, C E (1948). “A Mathematical Theory of Communication”. In: *Bell System Technical Journal* 27.3, pp. 379–423. DOI: 10.1002/j.1538-7305.1948.tb01338.x.

Stirparo, Giuliano G et al. (2018). “Integrated analysis of single-cell embryo data yields a unified transcriptome signature for the human pre-implantation epiblast”. In: *Development (Cambridge)* 145.3. DOI: 10.1242/dev.158501.
